# Strategies to Prevent Cholera Introduction during International Personnel Deployments: A Computational Modeling Analysis Based on the 2010 Haiti Outbreak

**DOI:** 10.1371/journal.pmed.1001947

**Published:** 2016-01-26

**Authors:** Joseph A. Lewnard, Marina Antillón, Gregg Gonsalves, Alice M. Miller, Albert I. Ko, Virginia E. Pitzer

**Affiliations:** 1 Department of Epidemiology of Microbial Diseases, Yale School of Public Health, New Haven, Connecticut, United States of America; 2 Global Health Justice Partnership, Yale University, New Haven, Connecticut, Untied States of America; 3 Yale Law School, New Haven, Connecticut, United States of America; 4 Department of Social and Behavioral Sciences, Yale School of Public Health, New Haven, Connecticut, United States of America; 5 Centro de Pesquisas Gonçalo Moniz, Fundação Oswaldo Cruz, Ministério da Saúde, Salvador, Brazil; Mahidol-Oxford Tropical Medicine Research Unit, THAILAND

## Abstract

**Background:**

Introduction of *Vibrio cholerae* to Haiti during the deployment of United Nations (UN) peacekeepers in 2010 resulted in one of the largest cholera epidemics of the modern era. Following the outbreak, a UN-commissioned independent panel recommended three pre-deployment intervention strategies to minimize the risk of cholera introduction in future peacekeeping operations: screening for *V*. *cholerae* carriage, administering prophylactic antimicrobial chemotherapies, or immunizing with oral cholera vaccines. However, uncertainty regarding the effectiveness of these approaches has forestalled their implementation by the UN. We assessed how the interventions would have impacted the likelihood of the Haiti cholera epidemic.

**Methods and Findings:**

We developed a stochastic model for cholera importation and transmission, fitted to reported cases during the first weeks of the 2010 outbreak in Haiti. Using this model, we estimated that diagnostic screening reduces the probability of cases occurring by 82% (95% credible interval: 75%, 85%); however, false-positive test outcomes may hamper this approach. Antimicrobial chemoprophylaxis at time of departure and oral cholera vaccination reduce the probability of cases by 50% (41%, 57%) and by up to 61% (58%, 63%), respectively. Chemoprophylaxis beginning 1 wk before departure confers a 91% (78%, 96%) reduction independently, and up to a 98% reduction (94%, 99%) if coupled with vaccination. These results are not sensitive to assumptions about the background cholera incidence rate in the endemic troop-sending country. Further research is needed to (1) validate the sensitivity and specificity of rapid test approaches for detecting asymptomatic carriage, (2) compare prophylactic efficacy across antimicrobial regimens, and (3) quantify the impact of oral cholera vaccine on transmission from asymptomatic carriers.

**Conclusions:**

Screening, chemoprophylaxis, and vaccination are all effective strategies to prevent cholera introduction during large-scale personnel deployments such as that precipitating the 2010 Haiti outbreak. Antimicrobial chemoprophylaxis was estimated to provide the greatest protection at the lowest cost among the approaches recently evaluated by the UN.

## Abstract 2

A cholera outbreak modeling study reveals that chemprophylaxis treatment for deployed peacekeepers in Haiti would have reduced the risk of disease introduction by 90%.

## Editors' Summary

**Background:**

Cholera—a bacterial gut infection caused by *Vibrio cholerae*—is a global killer. Epidemics (outbreaks) of cholera in countries across Africa, Asia, and South and Central America where cholera is endemic (always present) affect 1–4 million people and kill up to 142,000 people every year. People get cholera by eating food or drinking water contaminated with feces from an infected person, so cholera epidemics occur in places with poor sanitation such as refugee camps and in regions where an earthquake or another natural disaster has disrupted water and sanitation systems. Most people who become infected with *V*. *cholerae* have no or mild symptoms, but these asymptomatically infected individuals can shed bacteria in their feces for up to 2 wk. Other infected people develop severe diarrhea, producing profuse watery feces. The standard treatment for cholera is replacement of fluids and salts lost through diarrhea with oral rehydration fluid or, in the worst cases, by fluid replacement directly into a vein. If left untreated, patients with severe cholera can die from dehydration within hours of developing symptoms.

**Why Was This Study Done?:**

In 2010, a large cholera epidemic affected Haiti, which had been free of cholera for more than a century. This epidemic, which has resulted in nearly 9,000 deaths to date, was probably caused by contamination of water by infected sewage from the MINUSTAH (Mission des Nations Unies pour la stabilisation en Haïti) base, a UN peacekeeping mission established in Haiti in 2004. In January 2010, a massive earthquake hit Haiti, and in October that year, 454 troops from Nepal (a cholera-endemic country) were deployed to a MINUSTAH base in central Haiti as part of an ongoing peacekeeping operation to maintain political stability. Following the Haiti cholera outbreak, a panel of experts recommended that peacekeeping troops coming from countries where cholera is endemic should be screened for *V*. *cholerae* carriage, treated prophylactically with antimicrobial drugs (prophylactic treatments prevent a disease occurring), and/or immunized with oral cholera vaccines before deployment. However, because of uncertainty about the effectiveness of these pre-deployment intervention strategies, the UN has not implemented any of them. Here, the researchers use computational modeling to investigate whether these interventions would have prevented the Haiti cholera outbreak.

**What Did the Researchers Do and Find?:**

The researchers developed a mathematical model to simulate the arrival of asymptomatically infected peacekeepers (or those with mild illness that would not have prevented their deployment) and the dynamics of cholera transmission from the MINUSTAH base to the community. They used this model to investigate the effect of the three proposed pre-deployment intervention strategies on (1) the probability of undetected importation of *V*. *cholerae* from an endemic source country by an asymptomatically infected peacekeeper, and (2) the subsequent probability of transmission of *V*. *cholerae* from peacekeepers to the general public, two events needed to establish an epidemic. According to the model, diagnostic screening reduced the probability of cases occurring by 82%, whereas antimicrobial chemoprophylaxis at the time of departure and oral cholera vaccination reduced the probability of cases by 50% and 61%, respectively. Chemoprophylaxis beginning a week before deployment reduced the probability of cases by 91% when used alone and by 98% when coupled with vaccination. Finally, the researchers estimated that antimicrobial chemoprophylaxis with conventional therapies would cost under US$1 per peacekeeper, whereas the other two pre-deployment intervention strategies would be considerably more expensive.

**What Do These Findings Mean?:**

The accuracy of these findings is likely to be affected by the many assumptions made by the researchers in constructing their mathematical model. In addition, further research is needed to validate the use of rapid tests for detecting asymptomatic *V*. *cholerae* carriage, to compare the prophylactic efficacy of different antimicrobial regimens, and to quantify the impact of oral cholera vaccination on disease transmission from asymptomatic carriers. Nevertheless, these findings, which are likely to be generalizable to other settings, suggest that screening, chemoprophylaxis, and vaccination would all effectively prevent cholera introduction during large-scale personnel deployments like the one that precipitated the cholera outbreak in Haiti. However, although antimicrobial chemoprophylaxis is likely to provide the greatest protection at the lowest cost, this strategy is controversial because of increasing levels of drug resistance. Moreover, the researchers stress that proper sewage disposal must be implemented in tandem with any biomedical intervention designed to limit the risk of cholera introduction into disease-free areas by peacekeepers or other troops.

**Additional Information:**

This list of resources contains links that can be accessed when viewing the PDF on a device or via the online version of the article at http://dx.doi.org/10.1371/journal.pmed.1001947.

- The World Health Organization provides information about cholera in several languages
- The US Centers for Disease Control and Prevention also provides information about cholera for the public, medical professionals and travelers, specific information about the cholera epidemic in Haiti published a year into the outbreak, and CDC videos about defeating cholera and managing dehydration (in English, French and Spanish) are available
- The UK National Health Service (NHS) Choices website provides information about cholera
- Information about the UN in Haiti is available, including information on fighting cholera in the country, a recent fact sheet about the ongoing cholera epidemic, and a warning that cholera eradication in Haiti will take many years
- The United Nations has published the report of the Independent Panel of Experts on the Cholera Outbreak in Haiti and its own follow-up to the panel’s policy recommendations
- The not-for-profit organization Médecins Sans Frontières (MSF) is tackling several cholera outbreaks around the world; its website includes a 2014 report about the cholera epidemic in Haiti
- MedlinePlus provides links to further resources about cholera

## Introduction

An estimated 1.4 billion people are at risk for cholera in countries across Africa, Asia, and South and Central America where transmission is endemic [1]. In addition, explosive epidemics can occur when cholera is introduced to non-endemic populations. One of the most severe cholera epidemics of the modern era began in Haiti in 2010, causing over 700,000 reported cases and nearly 9,000 deaths to date [2]. Prior to the outbreak, cholera had been absent from Haiti for over a century [3]. Several pieces of evidence have contributed to widespread acceptance that the epidemic resulted from contamination of the Artibonite watershed with infected sewage from a United Nations Peacekeeping Mission in Haiti (MINUSTAH) base [4]. The causative *Vibrio cholerae* strain was imported from Nepal and diverged from strains circulating in that country around the time 454 Nepalese troops were deployed to Haiti [5], and the first cholera cases in Haiti were seen downstream from the base days after troops arrived [6].

Although preventing *V*. *cholerae* introduction is paramount for avoiding epidemics, there are no established protocols utilizing biomedical interventions to prevent cholera importation from endemic settings. An independent expert report to the United Nations (UN) following the Haiti outbreak [7] advocated for three pre-deployment interventions to limit transmission risk from peacekeepers: *V*. *cholerae* diagnostic screening, prophylaxis with antimicrobial chemotherapies, and immunization using oral cholera vaccines (OCVs). Diagnostic screening and antimicrobial chemoprophylaxis (a controversial control strategy amidst emerging drug resistance [8,9]) aim to decrease the risk of an infected person traveling and shedding *V*. *cholerae* on arrival. Antimicrobial drugs can also provide indirect protection against transmission by hastening bacterial clearance or preventing infection among individuals exposed to *V*. *cholerae*. OCVs, similarly, confer indirect protection by reducing bacterial output in stool [10,11].

Uncertainty about the extent to which these approaches reduce the risk for epidemic introduction is a contributing factor in the recent decision by the UN against implementing the recommended interventions [12,13]. To provide support for policymakers weighing the benefits of different approaches, we estimated how the proposed interventions would have influenced the likelihood of the 2010 cholera epidemic in Haiti, compared the costs associated with their implementation, and assessed how the various approaches would benefit peacekeepers by reducing their likelihood of suffering cholera.

## Methods

### Study Design

We compared interventions according to the probability for a symptomatic cholera case to occur in the host community. To estimate this, we developed a stochastic model simulating the arrival of asymptomatically infected peacekeepers (or those with mild illness that would not prevent them from being deployed) and dynamics of cholera transmission from the MINUSTAH base to the community under protocols in place as of October 2010. We used the model to assess the extent to which the proposed interventions alter the likelihood and dynamics of an introduced epidemic. Specifically, we quantified the impact of the interventions on the probability for two events requisite to the establishment of an epidemic: (1) undetected importation of *V*. *cholerae* from the endemic source country (Nepal) to Haiti by an asymptomatically infected peacekeeper and (2) transmission of *V*. *cholerae* from peacekeepers to the general public.

We provide an expanded description of the methods, including relevant equations, in S1 Text. R (version 3.2.1) scripts for implementing the model and related analyses are publicly available at https://github.com/joelewnard/choleraHaiti.git.

### Probability of Cholera Importation

#### Background incidence rate

The number of peacekeepers who were infected upon arrival at the MINUSTAH base is unknown. We inferred probability distributions for the prevalence of asymptomatic *V*. *cholerae* infection and incubation among peacekeepers at time of deployment based on background incidence rates reflecting potential transmission exposures experienced during their 10-d leave period preceding deployment (S1 Text §1.1). The annual, underreporting-adjusted incidence of cholera among Nepalese adults was previously estimated to be 1.8 cases per 1,000 persons [1]. In view of uncertainty associated with such estimates resulting from seasonal and geographic variation in incidence, we additionally modeled scenarios considering background incidence rates of 0.5, 1, 2, 5, and 10 cases annually per 1,000 persons.

#### Estimating symptom probability in an endemic setting

We inferred the prevalence of asymptomatic infection among peacekeepers via a meta-analysis of six epidemiological field studies that monitored the onset of symptomatic and asymptomatic *V*. *cholerae* shedding among household and community contacts of cholera patients (S1 Text §1.2) [14–19]. Since studies reported the number of contacts who experienced symptomatic and asymptomatic infections, the Beta distribution provided a direct way to compare study-level symptom probabilities based on available data; for each study *i*, we took the symptom probability *σ*_*i*_ to be Beta-distributed with the parameters *α*_*i*_ and *β*_*i*_ representing the number of new symptomatic and asymptomatic *V*. *cholerae* infections reported among contacts of index cases. The expected values and variances of the log-transformed symptom probability for each study were obtained as

$$E\left[\text{ln}\left(\sigma_{i}\right)\right]=\psi \left(\alpha_{i}\right)-\;\psi \left(\alpha_{i}+\beta_{i}\right)$$

for the digamma function *ψ*(*x*), and

$$V\left[\text{ln}\left(\sigma_{i}\right)\right]=\;\psi_{1}\left(\alpha_{i}\right)-\;\psi_{1}\left(\alpha_{i}+\beta_{i}\right)$$

for the trigamma function *ψ*_1_(*x*). We pooled the log-transformed means in an inverse variance-weighted random effects model to fit the distribution of the population parameter ln(*σ*) across studies using the metafor (version 1.9–4) package in R [20]. This approach resulted in an estimate that 24.2% (95% credible interval [CrI]: 14.4%, 40.7%) of cholera infections in an endemic setting were symptomatic (S1 Table).

#### Modeling importation

The peacekeeping battalion departed Nepal on October 7, arriving in Haiti on October 8 and entering the MINUSTAH base on October 9 [6,7]. We accounted for natural clearance of infection and intervention effects over the 2 d peacekeepers were in transit when estimating the prevalence of infection upon arrival at the MINUSTAH base. We modeled the time spent incubating and shedding *V*. *cholerae* as exponentially-distributed random variables. The probability for an individual peacekeeper to shed *V*. *cholerae* following arrival was equivalent to the probability that clearance had not occurred during transit, conditioned on having departed while incubating or asymptomatically shedding *V*. *cholerae* (S1 Text §1.3, 1.4).

We calibrated the model to replicate epidemiological observations following the arrival of differing numbers of infected peacekeepers, and we pooled outcome estimates according to a binomial probability distribution for each initial number of infected peacekeepers under the assumed background cholera incidence rates (S1 Text §1.1).

### Model Outline

Following peacekeepers’ arrival at a MINUSTAH base that disposed untreated sewage into a tributary of the Artibonite River, the early spread of cholera in central Haiti was mediated by downstream transport of *V*. *cholerae* to the lower Artibonite basin. The majority of early cases in this area were among individuals who farmed in rice fields adjacent to the Artibonite, drank untreated water from the river and its canals, and practiced open defecation [6,21,22]. Traditional routes of local interpersonal spread facilitated slower dissemination of the epidemic beyond the Artibonite basin [6,7]. Consequently, we modeled *V*. *cholerae* transmission via river-mediated and local pathways, partitioning the population of Haiti into several groups according to transmission exposures. The first group included persons in Artibonite River-adjacent communes situated downstream from the MINUSTAH base, among whom we considered a small subgroup coming into direct contact with the river for water and sanitation needs. The remaining population of Haiti, among whom we assumed no Artibonite exposure, was affected only by local transmission.

**Fig 1:**
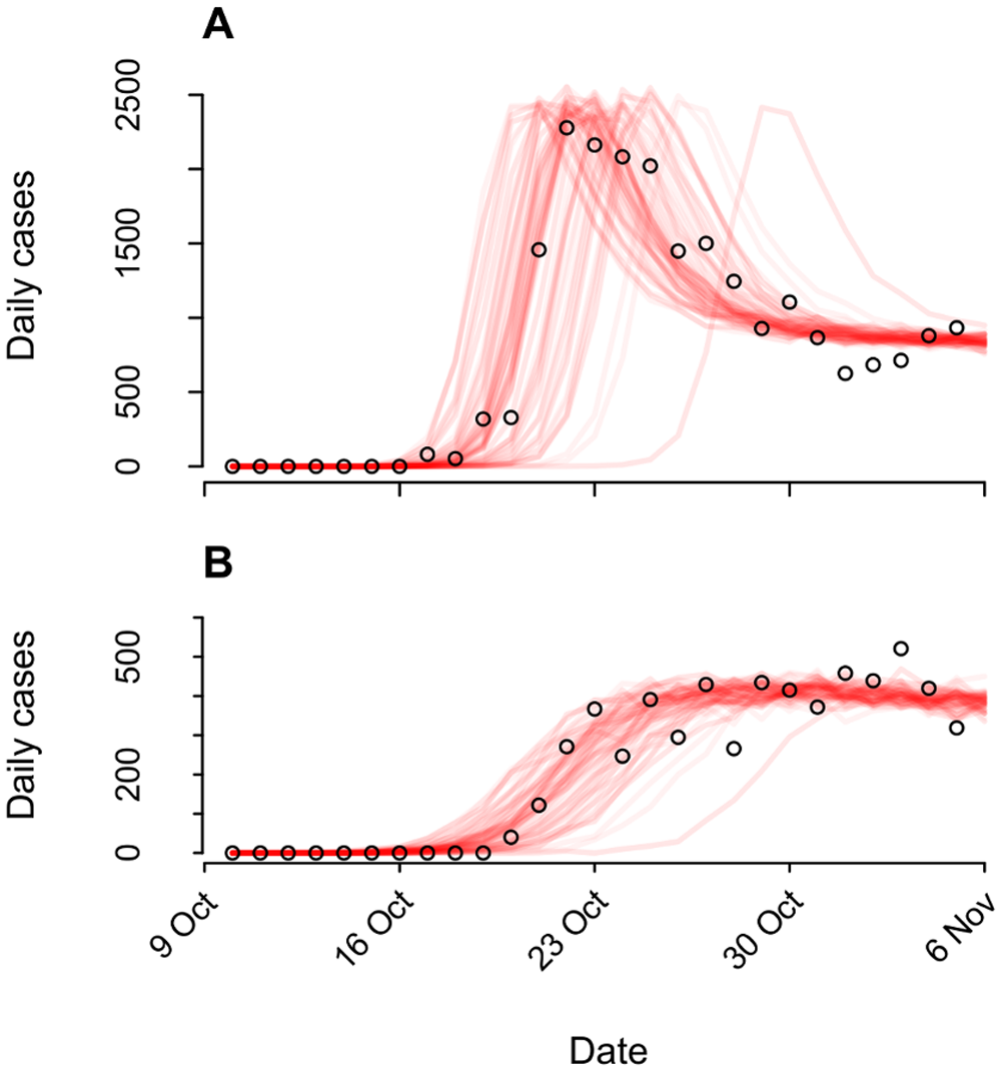
Epidemiological dynamics. We illustrate model-data concordance by plotting case observations (points) along with sample paths from stochastic realizations of the model (*n =* 100) under the status-quo scenario used for fitting. We plot instances (*n* = 79) in which transmission ensued and omit those where no epidemic occurred (*n* = 21). (**A**): Cases in the Artibonite-adjacent communes; (**B**): cases in all other communes.

Our model tracked a population of susceptible, latently infected, infectious, and recovered persons, as well as an environmental reservoir of *V*. *cholerae* in the Artibonite watershed (S1 Text §3.1). We used a quantitative dose-response relation (S1 Text §3.2, 3.4) linking *V*. *cholerae* exposure to individuals’ likelihood for infection and symptoms to account for variability in the distribution of symptomatic cases during cholera epidemics [19], exemplified in the Haiti epidemic by the precipitous rise in symptomatic cases 2 wk following peacekeepers’ arrival (Fig 1).

The relative infectiousness of symptomatic and asymptomatic cases in the context of local transmission is a source of uncertainty in cholera modeling [23,24]. Consequently, we calibrated the model using different log-linear relationships between stool output and infectiousness and verified that outcomes were not sensitive to our assumptions (S1 Text §3.4).

### Model Calibration

We derived model parameters for cholera natural history and disease based on data from clinical and epidemiological studies (Table 1). We propagated uncertainty in remaining parameters by sampling from possible values using a Bayesian Markov Chain Monte Carlo (MCMC) approach, calibrating model output to data on suspected and confirmed cholera cases from all ambulatory patients, hospital admissions, and deaths in Haiti from 16 October to 5 November 2010 (Table 2; S1 Text §4). The time period comprised the initial outbreak and its immediate spread within and outside the Artibonite Valley, prior to a second phase in the epidemic associated with transmission increases attributed to Hurricane Tomas [25,26].

**Table 1:** Fixed model parameters for cholera progression.

Parameter^a^	Definition	Value	Source
1/*δ*	Mean incubation period (d)	1.55	[27]
1/*γ*_*A*_	Expected duration of carriage (d)	5.09	[28,29]
1/*γ*_*D*_	Expected duration of diarrhea (d)	3.32	[28–34]
1/*γ*_*C*_	Expected duration of convalescent phase	1.77	[30]
*η*	Hyperinfectivity multiplier	100	[35–37]
*ν*	Diarrheal relative stool volume (*symptomatic case relative to asymptomatic carrier*)	8.58	[33,38–40]
*ξ*	Daily *V*. *cholerae* bacterial output	1^b^	(Fixed)
*κ*	*V*. *cholerae* dose with 50% infection probability	0.1^b^	(Fixed)
*ζ*	Case fatality rate	2.5%	[6]
Σ_*J*_ *N* ^(J)^	Population of Haiti (2010)	9,923,243	[41]
*N* ^(A)^	Population of Artibonite-adjacent communes (2010)	879,644	[6,41]

^a^Parameters’ relation to cholera natural history and transmission is detailed S1 Text §3.1.

^b^Estimates for the cholera infectious dose have varied widely in volunteer challenge trials due to host-level factors as well as whether individuals ingested *V*. *cholerae* with or without bicarbonate buffer solution, precluding numerical interpretation of the relation between the environmental exposure rate (*β*_*W*_), *ξ*, and *κ*. As a flexible alternative to previous models that sought to quantify *V*. *cholerae* exposure directly, we fixed *ξ* and *κ* as numerical constants and estimated *β*_*W*_ values corresponding to exposure levels requisite for inducing infections as they were observed in Haiti.

**Table 2:** Estimated parameters for transmission dynamics in Haiti.

Parameter	Definition	Prior	Source	Estimate^a^,^b^
*π*	Symptom probability (*local transmission*)	Beta(263, 812)^c^	[42,43]	0.241 (0.219, 0.267)
Log_10_(*β*_*W*_)	Environmental *V*. *cholerae* exposure rate	Unif(–∞, –3)	[23,44]	–6.665 (–6.676, –6.653)^d^
*β*_*L*_	Contact rate with *V*. *cholerae* carriers	Unif(0.1, 0.6)		0.219 (0.184, 0.260)
*k*	Scaling constant for local transmission^e^	Unif(0, 10^6^)		1201 (1010, 1413)
Log_10_(*ω*)	Migration rate (d^–1^)	Unif(–2,–0.3)		–1.314 (–1.331, –1.29)^f^
Log_10_(*N* ^(W)^/*N* ^(A)^)	Proportion exposed to Artibonite (*of Artibonite-adjacent communes*)	Unif(–3, –1)	[6,44]	–1.618 (–1.630, –1.608)^g^

^a^Estimates are presented for the case *r* = Log_10_(*ν*) and the initial condition of one peacekeeper arriving infected. Estimates for differing *r* and initial conditions are presented in S15 Table.

^b^Estimates are reported as median (95% CrI) describing the distribution generated by Markov Chain Monte Carlo sampling.

^c^The informative prior applied for estimating the symptom probability results from a serological survey measuring symptom probabilities during the initial outbreak in the study region [43] (*n =* 948) and a challenge study estimating the probability for cholera-naïve individuals to experience symptoms when exposed to *V*. *cholerae* [42] (*n* = 127), in contrast to the symptom probability for endemic settings estimated in S1 Table (S1 Text §4.2).

^d^Exponentiated: (2.161 (2.108, 2.226)) × 10^−7^ per bacterial unit per day (see S1 Text §3.2).

^e^Smaller values represent more clustered local transmission, while larger values represent more homogeneous mixing (see S1 Text §3.2).

^f^Exponentiated: 0.0485 (0.0467, 0.0507) per day.

^g^Exponentiated: 0.0241 (0.0234, 0.0247)

### Interventions

**Fig 2:**
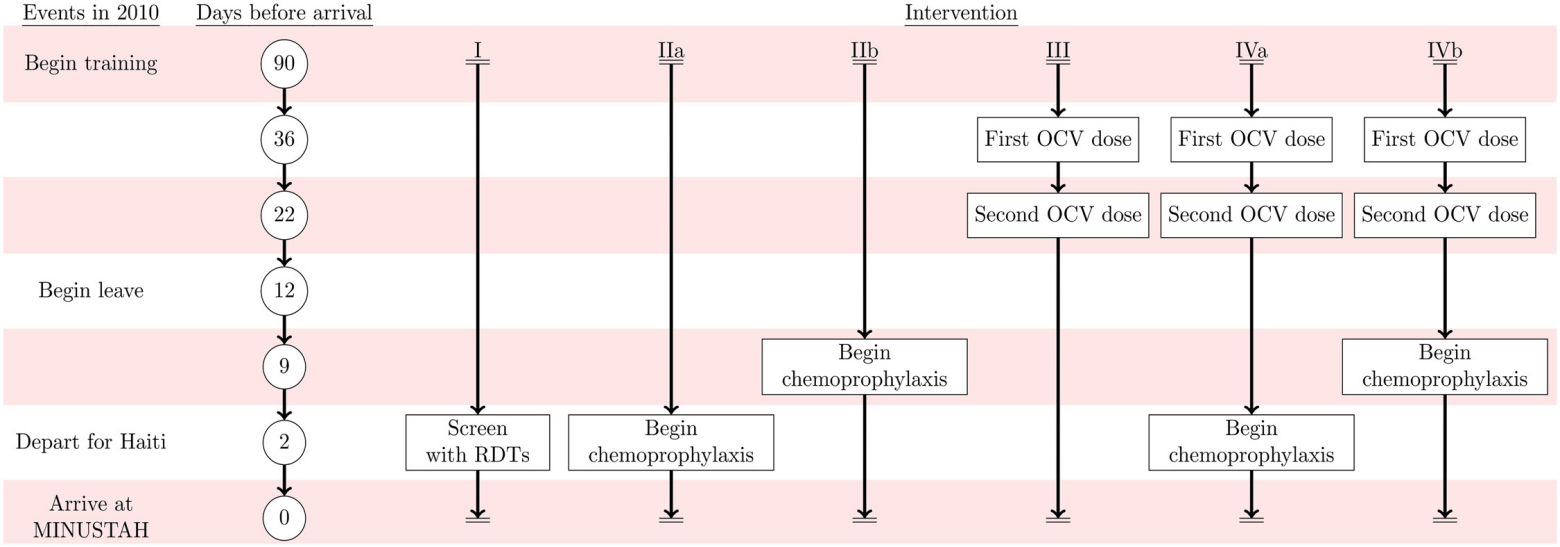
Intervention timeline. Events in 2010 are listed on the left alongside the timeframe for the simulated interventions: (I) RDT screening, (IIa) time-of-departure prophylaxis with antimicrobial drugs, (IIb) early-initiated prophylaxis with antimicrobial drugs beginning 7 d prior to deployment, (III) two-dose OCV immunization at 36 and 22 d prior to deployment, (IVa) two-dose OCV immunization combined with time-of-departure chemoprophylaxis, and (IVb) two-dose OCV immunization combined with early-initiated chemoprophylaxis.

We compared four interventions against status-quo protocols: (I) screening peacekeepers for *V*. *cholerae* carriage at their time of departure from Nepal using a licensed, commercially-available immunochromatographic rapid diagnostic test (RDT) following an enrichment step; (IIa) administering antimicrobial chemotherapy at time of departure (time-of-departure prophylaxis) or (IIb) beginning 7 d prior to departure (early-initiated prophylaxis); (III) immunizing peacekeepers using OCV administered beginning 5 wk before departure; and (IV) combined immunization and chemoprophylaxis following the time-of-departure (IVa) and early-initiated (IVb) schedules (Fig 2, Table 3).

**Table 3:** Individual-level intervention efficacy.

Parameter	Definition	Value^a^	Source
*Φ*	Relative infectiousness of OCV recipients (*relative to unvaccinated asymptomatic carriers*)	0.0097–0.5 (varied); 0.0194 (Main Text)	[11]
Se	RDT sensitivity (*enriched rectal swab method*)^b^,^c^	96.0% (89.4%, 99.1%)	[45]
Sp	RDT specificity (*enriched rectal swab method*)^b^,^c^	92.4% (85.0%, 97.5%)	[45]
*υ*_Abx_	Relative risk for cholera shedding (*cholera-exposed persons receiving prophylaxis relative to non-recipients*)	34.3% (18.1%, 65.3%)	[9]
1/*γ*_*A*_− 1/*γ*_Abx_	Reduction in duration of shedding (d) (*prophylaxed persons experiencing shedding relative to non-prophylaxed infected individuals*)	2.74 (2.40, 3.07)	[30]

^a^Values are presented as median (95% CrI) based on distributions measured in cited studies or inferred from the reported data. We propagated uncertainty in intervention efficacy by population simulations with Monte Carlo draws from the estimated or reported distributions.

^b^Test sensitivity and specificity are assumed to follow Beta distributions inferred by the approach described in S1 Text (§2.2).

^c^Sensitivity and specificity are calculated for the cholera O1 dipstick test and compared to culture as a gold standard.

We assessed the impact of the interventions on the probability for a symptomatic cholera case to occur in the host community in Haiti.

#### Status quo scenario

We assume the observed epidemic was representative of a status-quo scenario entailing no testing, antimicrobial chemotherapy, or immunization among asymptomatic peacekeepers, as per current (and 2010) pre-departure indications [7,12,13]. Existing protocols include the isolation and clinical management of diarrheal cases. We assumed that these measures are effective in limiting the risk for peacekeepers experiencing symptomatic cholera during deployment to transmit infection under status quo. We consequently modeled the impact of interventions on the probability for transmission by asymptomatically infected peacekeepers only.

#### Rapid diagnostic screening

Recommendations for the UN to screen departing peacekeepers are most likely to be implemented using rapid diagnostic tests (RDTs), which are capable of detecting *V*. *cholerae* at low densities obtainable via rectal swabs of asymptomatic carriers following brief (4–6h) enrichment [45,46]. Alternative methods for identifying *V*. *cholerae* among asymptomatic carriers, such as culture or polymerase chain reaction, may be impractical by comparison for routine peacekeeping operations because individuals may acquire or clear infection in the time required for diagnosis (24–72h), and/or because of limited laboratory facilities in many cholera-endemic settings [47]. We used previous sensitivity and specificity estimates for the leading licensed, commercially-available RDT (Crystal VC, Span Diagnostics, Surat, India) following an enrichment step (“confirmation method” [45]) to quantify the impact of screening on the probability for infected cholera-infected peacekeepers to be deployed (S1 Text §2.2). The confirmation method yields higher specificity than direct stool screening via the dipstick. We conducted further analyses considering sensitivities and specificities ranging from 50% to 99% to examine the range of plausible outcomes under different point-of-departure screening protocols.

#### Antimicrobial chemoprophylaxis

Numerous antimicrobial drugs are efficacious for cholera treatment and prophylaxis. We used estimates from meta-analyses [9,30] aggregated across antimicrobial drug classes to quantify two correlates of protection: (1) reduced risk for infection, and (2) shortened duration of shedding given infection (S1 Text §2.3). We considered two drug administration schedules. The first was a typical prophylaxis approach beginning at the time of departure from Nepal (IIa; “time-of-departure” prophylaxis), intended to hasten clearance of infection among peacekeepers who acquired infection during their leave period. Shown to be particularly effective in an early trial [48], the second (IIb; “early-initiated” prophylaxis) was to administer prophylaxis beginning 7 d prior to deployment, which would both hasten clearance and prevent *V*. *cholerae* infection in the days leading up to departure.

Emergence of resistance to conventional cholera therapies, such as tetracycline, doxycycline, and ciprofloxacin, undermines interpretation of antimicrobial drug efficacies reported in earlier trials [9,30]. We therefore assessed how externalities such as reduced antimicrobial susceptibility may limit the effectiveness of chemoprophylaxis interventions by conducting a sensitivity analysis, considering that conventional antimicrobial agents conferred 10% to 50% lower-than-reported efficacy. While resistance to azithromycin is less prevalent and single-dose regimens of this drug have shown superiority over conventional therapies for cholera treatment [30,49], the efficacy of azithromycin prophylaxis has not been studied. We also assessed the potential utility of azithromycin under a time-of-departure single-dose regimen assuming 10%–50% superior prophylactic efficacy over conventional therapies.

#### Oral cholera vaccination

Although OCVs do not necessarily protect recipients against cholera infection, they reduce density of *V*. *cholerae* in stool and reduce an individual’s likelihood for experiencing disease given that infection occurs [10,11]. We modeled reductions in bacterial shedding among asymptomatic peacekeepers resulting from immunization with two doses of killed bivalent whole-cell OCV (Shanchol, Shantha Biotechnics, Hyderabad, India), administered beginning 5 wk before departure, to compare transmission risk from immunized and non-immunized peacekeepers who experience asymptomatic infection (S1 Text §2.4). While numerical reductions in excreted *V*. *cholerae* density among OCV recipients have been reported in a challenge trial [11], there is uncertainty in the quantitative relation between total excreted *V*. *cholerae* and transmission risk [23]. We therefore conducted a sensitivity analysis comparing effectiveness estimates assuming differing levels of protection against transmission conferred by OCV.

Current and next-generation OCVs, including recombinant live vaccines (e.g., CVD 103-HgR), may confer some protection against infection in addition to reducing bacterial shedding among recipients [11,50,51]. Considering this possibility, we performed an additional sensitivity analysis considering potential outcomes of OCV interventions when vaccines were modeled to prevent a proportion (5%, 10%, 25%, or 50%) of recipients from experiencing infection, in addition to conferring reductions in shedding among recipients who become infected.

#### Combined antimicrobial chemoprophylaxis and immunization

We assessed the effectiveness of a combined intervention where peacekeepers received two doses of OCV beginning 5 wk before departure as well as either early-initiated or time-of-departure antimicrobial chemotherapy. Under such an intervention, peacekeepers’ likelihood for experiencing shedding upon arrival and rate of clearing *V*. *cholerae* infection were determined using the parameters for antimicrobial chemoprophylaxis interventions described above. Peacekeepers were assumed to shed *V*. *cholerae* at reduced densities due to vaccine receipt, as for the OCV interventions.

#### Benefit to peacekeepers

In addition to assessing how the interventions under consideration affect the probability for asymptomatic peacekeepers to import and transmit *V*. *cholerae*, we estimated how antimicrobial chemoprophylaxis and OCV immunization benefit peacekeeping forces by lowering individuals’ risk for experiencing symptomatic cholera disease. We estimated these reductions using outcomes of previous studies that assessed direct effects of antimicrobials and OCV in preventing (1) *V*. *cholerae* infection, and (2) cholera symptoms given that infection occurs [9,11,52].

#### Intervention costs

We estimated the direct costs of the interventions in terms of the cost per peacekeeper of purchasing the necessary tests, drugs, or vaccine doses, excluding additional indirect costs associated with delivery and implementation. We considered the most recently-reported manufacturer prices for RDTs and generic antibiotics [53]. We assessed OCV costs based on reported per-dose pricing for the global OCV stockpile [54].

### Model Implementation

To incorporate measures of uncertainty resulting from model parameterization and the stochastic nature of transmission, we implemented the model using the Gillespie algorithm (S1 Text §3.8), sampling fitted parameter values from their posterior distribution inferred by MCMC [55]. We propagated uncertainty in screening and chemoprophylaxis intervention efficacy via Monte Carlo sampling from the fitted distributions in stochastic model realizations. We computed probabilities for a symptomatic cholera case in the Haitian population (after which a large epidemic was likely to occur) as the proportion of model realizations in which a case occurred prior to the extinction of *V*. *cholerae* transmission. We calculated relative risk estimates comparing probabilities under intervention scenarios against status quo. Due to the computationally-intensive nature of the model, we generated 95% credible intervals surrounding effect size estimates via bootstrap resampling. We carried out computations on the Louise High-Performance Computing Cluster at Yale University.

## Results

### Infectious Arrivals

The estimated prevalence of asymptomatic *V*. *cholerae* infection and incubation among peacekeepers at their time of deployment ranged from 2.9 (1.3, 5.5) to 57.4 (26.2, 110.6) per 100,000 for background incidence rates of 0.5 to 10 cases per 1,000 person-years at risk (PYAR) (S2 Table). Allowing for cholera progression during transit to the MINUSTAH base, we estimated the prevalence of *V*. *cholerae* carriage among arrivals to range from 1.8 (0.8, 3.4) to 35.4 (16.2, 68.2) per 100,000 (S2 Table). These estimates corresponded to a probability of between 0.8% (0.4%, 1.6%) and 14.8% (7.0%, 26.9%), respectively, for at least one of the 454 peacekeepers to have been infected upon arrival at the MINUSTAH base (Table 4).

**Table 4:** Importation probabilities under status quo and intervention scenarios, with estimated effectiveness against importation.

Intervention	Outcome measure (%)^a^	Background cholera incidence rate					
		0.5/1,000 PYAR	1.0/1,000 PYAR	1.8/1,000 PYAR^b^	2.0/1,000 PYAR	5.0/1,000 PYAR	10.0/1,000 PYAR
Status quo	Probability	0.8 (0.4, 1.6)	1.6 (0.7, 3.1)	**2.9 (1.3, 5.4)**	3.2 (1.4, 6.1)	7.7 (3.6, 14.5)	14.8 (7.0, 26.9)
RDT screening	Probability	0.2 (0.1, 0.3)	0.3 (0.1, 0.6)	**0.5 (0.2, 1.1)**	0.6 (0.3, 1.2)	1.5 (0.7, 2.9)	2.9 (1.3, 5.8)
	Effectiveness	81.7 (76.1, 84.5)	81.7 (76.0, 84.5)	**81.6 (75.9, 84.4)**	81.5 (75.9, 84.4)	81.2 (75.4, 84.1)	80.5 (74.5, 83.6)
Time-of-departure prophylaxis	Probability	0.5 (0.2, 1.0)	1.0 (0.4, 1.9)	**1.7 (0.7, 3.7)**	1.9 (0.9, 3.8)	4.8 (2.2, 9.1)	9.3 (4.3, 17.4)
	Effectiveness	39.3 (30.9, 47.1)	39.2 (30.8, 47.0)	**39.0 (30.5, 46.9)**	39.0 (30.5, 46.9)	38.4 (29.7, 46.6)	37.4 (28.3, 46.0)
Early-initiated prophylaxis	Probability	0.1 (0.0, 0.3)	0.2 (0.1, 0.6)	**0.3 (0.1, 1.0)**	0.4 (0.1, 1.1)	0.8 (0.3, 2.8)	1.7 (0.5, 5.5)
	Effectiveness	89.1 (74.0, 95.0)	89.0 (74.0, 95.0)	**89.0 (73.8, 94.9)**	88.9 (73.8, 94.9)	88.7 (73.3, 94.8)	88.3 (72.3, 94.6)

PYAR: person-years at risk (incidence rate denominator).

^a^Reported probabilities refer to the likelihood that at least one peacekeeper in a battalion of 454 arrives experiencing asymptomatic infection after 2 d in transit from a source country with the designated background cholera incidence rate. Effectiveness is defined as the reduction in this probability relative to its estimate under status quo protocols. Estimates are reported as median (95% CrI), as obtained via the binomial distribution parameterized according to the procedures described in S1 Text §1.1.

^b^The incidence of cholera among adults in Nepal was previously estimated to be 1.8/1,000 PYAR [1].

### Epidemiological Dynamics

We estimated the basic reproductive number (*R*_0_) at the outset of the epidemic to have been 1.80 (1.64, 2.00) within Artibonite-adjacent communes and 1.13 (0.98, 1.34) nationwide assuming a background cholera incidence rate of 1.8 per 1,000 PYAR in Nepal (S1 Text §3.9) (Table 5). This value defined the number of secondary cases an index case was expected to cause in the fully susceptible population, and did not vary significantly across the incidence rates considered (Table 5) or among fitted models that assumed a different relationship between stool output and infectiousness (S13 Table). Quantifying the contributions of the modeled transmission pathways, we estimated the reproductive number for Artibonite-mediated *V*. *cholerae* transport was 30.24 (30.03, 30.44), while most transmission occurred via slower local spread with a reproductive number equal to 1.07 (0.91, 1.27). Cholera cases were expected to occur in the community with 82.2% (81.2%, 83.2%) probability following the arrival of one infected peacekeeper, and with over 95% probability following the arrival of two or more infected peacekeepers (Table 6).

**Table 5:** Estimated basic reproduction numbers (*R*_0_).

Initial conditions		Basic reproduction number (*R*_0_) estimate			
		Geographical area		Transmission pathway	
		Artibonite communes	Nationwide	River-mediated	Local
Number of infected arrivals^a^	1	1.80 (1.64, 2.01)	1.14 (0.97, 1.34)	30.29 (30.09, 30.50)	1.07 (0.91, 1.28)
	2	1.59 (1.44, 1.79)	0.97 (0.82, 1.17)	26.65 (26.48, 26.83)	0.91 (0.76, 1.11)
	3	1.48 (1.36, 1.66)	0.89 (0.77, 1.07)	24.68 (24.52, 24.83)	0.83 (0.71, 1.02)
Background cholera incidence rate^b^	0.5/1,000 PYAR	1.80 (1.64, 2.01)	1.14 (0.97, 1.34)	30.27 (30.07, 30.48)	1.07 (0.91, 1.28)
	1.0/1,000 PYAR	1.80 (1.64, 2.01)	1.14 (0.98, 1.34)	30.26 (30.06, 30.47)	1.07 (0.91, 1.28)
	**1.8/1,000 PYAR (WHO est.)** ^c^	**1.80 (1.64, 2.00)**	**1.13 (0.98, 1.34)**	**30.24 (30.03, 30.44)**	**1.07 (0.91, 1.27)**
	2.0/1,000 PYAR	1.80 (1.64, 2.00)	1.14 (0.98, 1.34)	30.23 (30.02, 30.44)	1.07 (0.91, 1.27)
	5.0/1,000 PYAR	1.79 (1.64, 1.99)	1.13 (0.97, 1.33)	30.14 (29.91, 30.36)	1.07 (0.91, 1.26)
	10.0/1,000 PYAR	1.79 (1.63, 1.97)	1.13 (0.97, 1.31)	29.99 (29.68, 30.25)	1.06 (0.91, 1.25)

PYAR: person-years at risk (incidence rate denominator).

^a^ *R*_0_ estimates are reported as median (95% CrI) from the posterior distribution of the parameters fitted under assumptions of one, two, or three infected peacekeepers at the outset of the epidemic. We derive the formula for *R*_0_ via the next-generation matrix approach of [56] (S1 Text §3.9).

^b^Incidence-rate-specific *R*_0_ estimates are obtained by pooling estimates across parameter sets according to the binomial probabilities of one, two, or three infected peacekeepers arriving (S1 Text §1.1).

^c^The incidence of cholera among adults in Nepal was previously estimated to be 1.8/1,000 PYAR [1].

**Table 6:** Case probabilities following importation by one, two, or three peacekeepers.

Intervention	Outcome measure (%)^a^,^b^	Infectious arrivals^c^		
		1	2	3
Status quo	Case probability	82.2 (81.1, 83.2)	95.9 (95.3, 96.4)	99.1 (98.9, 99.4)
Antimicrobial chemoprophylaxis (ACP)^d^	Case probability	67.5 (66.2, 68.8)	87.8 (86.8, 88.7)	95.4 (94.8, 96.0)
	Effectiveness	17.8 (16.0, 19.8)	8.4 (7.4, 9.5)	3.8 (3.2, 4.4)
Oral cholera vaccination (OCV)	Case probability	32.1 (30.8, 33.4)	49.9 (48.5, 51.3)	59.8 (58.5, 61.2)
	Effectiveness	61.0 (59.3, 62.7)	47.9 (46.4, 49.4)	39.6 (38.3, 41.0)
Combined ACP^d^ and OCV	Case probability	17.3 (16.3, 18.4)	29.7 (28.5, 31.0)	38.3 (36.9, 39.6)
	Effectiveness	78.9 (77.6, 80.2)	69.0 (67.7, 70.3)	61.4 (60.0, 62.7)

^a^The case probability outcome measure is defined as the likelihood for at least one symptomatic cholera case to occur among members of the host community following arrival of one, two, or three infected peacekeepers.

^b^Effectiveness is defined as the reduction in this probability relative to its estimate under status quo.

^c^Estimates are reported as median (95% CrI), as obtained via bootstrap resampling.

^d^Estimates apply to both early-initiated and time-of-departure chemoprophylaxis schedules; the rate at which prophylaxed individuals clear infection is the same under both schedules, whereas the interventions differ in probabilities for arrival of one, two, or three infected peacekeepers

Pooling case probabilities according to the likelihood for one, two, or three peacekeepers arriving infected (S3 Table; S1 Text §1.1), we estimated the total probability for community cases ranged from 0.7% (0.3%, 1.3%) to 12.4% (5.8%, 22.4%) under the different background incidence rates modeled. This outcome did not depend on the modeled relationship between shedding rates and infectiousness among symptomatic and asymptomatic peacekeepers (S14 Table). The timing of the first symptomatic case was subject to variability across stochastic realizations of the model (95% CrI: October 11 to October 20) (Fig 1). The first symptomatic case is reported to have occurred October 12 [57]; our model predicts a case on or before this date in 24% of simulations, whereas 71% of simulations predict a case on or before October 14, which is the date of symptoms onset for the first culture-confirmed cholera cases identified by a Cuban medical brigade that investigated the outbreak early in its course (S1 Text §4.5) [6].

### Intervention Effects

Using RDTs with an enrichment step to screen for and exclude potential cholera carriers from transiting to Haiti conferred an 81.6% (75.9%, 84.4%) reduction in the probability for *V*. *cholerae* importation at the assumed background incidence rate of 1.8 cases per 1,000 PYAR (Table 4, Table 7). However, under such a screening program, 7% (2%, 15%) of tests administered (33 [10, 70] peacekeepers in a battalion of 454) were expected to result in false positive outcomes. Reductions in importation probabilities and expected false-positive rates did not vary significantly across the range of background incidence rates considered (S4 and S5 Tables). A point-of-departure screening approach with 99% sensitivity and specificity would reduce the importation probability by up to 83% relative to status quo, with 0.9% of tests (4 of 454) returning false-positive results.

**Table 7:** Total reduction in case probability afforded by interventions, with estimated intervention effectiveness.

Outcome measure (%)^a^,^b^	Background cholera incidence rate	Status quo	Rapid diagnostic testing (RDT)	Antimicrobial chemoprophylaxis (ACP)		Oral cholera vaccine (OCV)	Combined ACP and OCV	
				Time-of-departure	Early-initiated		Time-of-departure	Early-initiated
Case probability	0.5/1000 PYAR	0.7 (0.3, 1.3)	0.1 (0.1, 0.2)	0.3 (0.1, 0.7)	0.1 (0.0, 0.2)	0.3 (0.1, 0.5)	0.1 (0.0, 0.2)	0.02 (0.004, 0.05)
	1.0/1000 PYAR	1.3 (0.6, 2.5)	0.2 (0.1, 0.5)	0.7 (0.3, 1.4)	0.1 (0.0, 0.4)	0.5 (0.2, 1.0)	0.2 (0.1, 0.4)	0.03 (0.01, 0.1)
	**1.8/1000 PYAR** ^**c**^	**2.3 (1.1, 4.5)**	**0.4 (0.2, 0.9)**	**1.2 (0.5, 2.5)**	**0.2 (0.1, 0.7)**	**0.9 (0.4, 1.8)**	**0.3 (0.1, 0.6)**	**0.1 (0.0, 0.2)**
	2.0/1000 PYAR	2.6 (1.2, 5.0)	0.5 (0.2, 1.0)	1.3 (0.5, 2.8)	0.2 (0.1, 0.8)	1.0 (0.5, 2.0)	0.3 (0.1, 0.7)	0.1 (0.0, 0.2)
	5.0/1000 PYAR	6.4 (3.0, 11.9)	1.2 (0.5, 2.4)	3.2 (1.3, 6.7)	0.6 (0.2, 1.9)	2.5 (1.2, 4.8)	0.8 (0.3, 1.8)	0.2 (0.0, 0.5)
	10.0/1000 PYAR	12.4 (5.8, 22.4)	2.4 (1.1, 4.8)	6.4 (2.6, 13.0)	1.2 (0.3, 3.8)	5.0 (2.3, 9.2)	1.7 (0.7, 3.5)	0.3 (0.1, 1.0)
Effectiveness	0.5/1000 PYAR		81.7 (76.1, 84.5)	50.1 (43.1, 56.5)	91.0 (78.7, 95.9)	60.9 (59.3, 62.6)	87.2 (85.2, 89.0)	97.7 (94.5, 98.9)
	1.0/1000 PYAR		81.7 (76.1, 84.5)	50.0 (42.9, 56.5)	91.0 (78.6, 95.9)	60.9 (59.2, 62.5)	87.2 (85.2, 89.0)	97.7 (94.5, 98.9)
	**1.8/1000 PYAR** ^c^		**81.6 (76.0, 84.4)**	**49.9 (42.7, 56.4)**	**91.0 (78.5, 95.9)**	**60.8 (59.1, 62.4)**	**87.1 (85.1, 88.9)**	**97.7 (94.5, 98.9)**
	2.0/1000 PYAR		81.6 (75.9, 84.4)	49.8 (42.7, 56.4)	90.9 (78.5, 95.8)	60.7 (59.1, 62.4)	87.1 (85.0, 88.9)	97.7 (94.5, 98.9)
	5.0/1000 PYAR		81.3 (75.6, 84.2)	49.3 (41.9, 56.2)	90.8 (78.1, 95.8)	60.4 (58.7, 62.0)	87.9 (84.7, 88.8)	97.6 (94.3, 98.9)
	10.0/1000 PYAR		80.7 (74.8, 83.7)	48.5 (40.6, 55.8)	90.4 (77.5, 95.7)	59.7 (58.0, 61.5)	86.5 (84.1, 88.6)	97.6 (94.1, 98.9)

PYAR: person-years at risk (incidence rate denominator).

^a^Case probabilities refer to the likelihood that at least one symptomatic cholera case occurs in the community. Effectiveness is defined as the reduction in this probability relative to its estimate under status quo protocols.

^b^Estimates are reported as median (95% CrI), as obtained via bootstrap resampling.

^c^The incidence of cholera among adults in Nepal was previously estimated to be 1.8/1000 PYAR [1].

Early-initiated antimicrobial chemoprophylaxis offered marginally superior protection against importation, reducing the probability of at least one infected peacekeeper arriving by 89.0% (73.8%, 94.9%). In contrast, prophylaxis delivered at time of departure reduced the importation probability by only 39.0% (30.5%, 46.9%) (Table 4). Accounting for both protection against importation as well as reduced opportunities for transmission due to faster clearance of infection among treated peacekeepers, antimicrobial chemoprophylaxis was expected to reduce the probability for a case by 49.9% (42.7%, 56.4%) when administered at time of departure, or by 91.0% (78.5%, 95.9%) if treatment were initiated 1 wk before departure (Table 7).

OCV immunization reduced the probability for a case in the community by up to 60.8% (59.1%, 62.4%). Concurrent OCV immunization and antimicrobial chemoprophylaxis resulted in up to 87.1% (85.1%, 88.9%) and 97.7% (94.5%, 98.9%) reductions in the probability for a case under time-of-departure and early-initiated treatment regimens, respectively.

We found that the effectiveness of immunization was highly sensitive to the modeled level of protection resulting from OCV-mediated reductions in shedding. If vaccination reduces the risk of transmission from an asymptomatically infected individual by less than 96% (*ϕ* = 0.0388), OCV interventions cease to be more effective than time-of-departure chemoprophylaxis. Even assuming reductions in transmission risk greater than 99% (*ϕ* = 0.0097), OCV interventions offered lower effectiveness than screening and early-initiated prophylaxis (S7 Table). At reported levels of reductions in bacterial shedding (*ϕ* = 0.0194) [11], OCV interventions offered comparable effectiveness (>80% reduction in case probability) to screening and early-initiated prophylaxis only if we assumed vaccination reduces the probability of any infection by 50% (S16 Table).

In contrast, antimicrobial chemoprophylaxis remained effective when considering potential limitations that could undermine this approach. Early-initiated chemoprophylaxis showed superior effectiveness over RDT screening when we assumed up to 25% lower-than-reported efficacy of antimicrobial drugs, as might be expected if resistance to first-line chemotherapies or poor compliance with drug regimens limited this approach (S6 Table). Early-initiated chemoprophylaxis remained superior to OCV interventions even when we assumed 50% lower-than-reported efficacy for antimicrobial drugs. Under such conditions, we expected a 69.7% (34.6%, 85.1%) reduction in the probability for a symptomatic cholera case.

Administering a single dose of azithromycin at time of departure was expected to offer superior effectiveness over screening and early-initiated prophylaxis with conventional therapies if azithromycin offers a 50% improvement in protection against shedding onset and a 50% greater reduction in the duration of shedding relative to conventional drugs (S8 Table). Even if the efficacy of azithromycin is only 10% superior to conventional agents, time-of-departure prophylaxis with this treatment offers a 56.6% (49.3%, 64.1%) reduction in the probability for a case, nearly equal to the estimated effectiveness of OCV.

### Benefit to Peacekeepers

Antimicrobial chemoprophylaxis and immunization with OCV directly benefit peacekeepers from cholera-endemic settings by reducing their risk for experiencing symptomatic cholera illness. These benefits would also be afforded to peacekeepers traveling to endemic countries where they may be exposed to cholera, with OCV offering longer-lasting protection. Based on available studies (Table 8), we estimated that antimicrobial drugs lower the probability for experiencing cholera diarrhea by 95.5% (70.4%, 99.9%) among peacekeepers exposed to *V*. *cholerae* before deployment. OCV interventions are expected to reduce exposed peacekeepers’ probability for cholera diarrhea by 62.8% (22.2%, 90.0%). Combining chemoprophylaxis with vaccination provides up to 98.0% (84.9%, 99.9%) protection against cholera diarrhea.

**Table 8:** Benefit to peacekeepers.

	Reduction in probability of cholera diarrhea (%)^a^	Source
Antimicrobial chemoprophylaxis (ACP)	95.5 (70.4, 99.9)	[9,52]
Oral cholera vaccine (OCV)	62.8 (22.2, 90.0)	[11]
Combined ACP and OCV	98.0 (84.9, 99.9)	[9,11,52]

^a^Estimates are reported as median (95% CrI) as estimated by the approach described in S1 Text (§2.5).

### Intervention Costs

Antimicrobial chemoprophylaxis is the lowest-cost strategy under consideration (Table 9). Early-initiated and time-of-departure chemoprophylaxis with conventional cholera therapies each cost under US$1 per peacekeeper, while single-dose prophylaxis using azithromycin would cost between US$0.52 and US$1.32 per peacekeeper. Although the direct cost of purchasing RDTs is only moderately more expensive at US$2.54 per peacekeeper, there are additional indirect costs associated with training and employing personnel to take rectal swabs, perform sample enrichment, and administer tests, which together may exceed the direct costs of assays [58]. Delaying the deployment of peacekeepers who test positive for *V*. *cholerae* imposes additional indirect costs and logistical burden, particularly if tests have low specificity (S5 Table). Administering OCV is expected to cost US$3.70 per peacekeeper for the bivalent vaccine without B subunit (Shancol), or between US$9.40 and US$18.80 for the vaccine containing B subunit (Dukoral). Additional indirect costs associated with maintaining a cold chain for shipment and storage of the vaccine [54] are not reflected in the unit price of vaccine doses.

**Table 9:** Intervention costs.

Intervention	Method	Direct cost per peacekeeper^a^,^b^	Indirect costs and considerations
Rapid diagnostic screening	Crystal VC cholera dipstick (Span Diagnostics, Surat, India)	US$2.54 [59]	Direct costs do not include the cost of laboratory personnel to perform sample enrichment and rectal swabbing, which are likely to exceed the cost of assays [58]. Additional costs or logistical difficulties may result from preventing deployment of peacekeepers with *V*. *cholerae*-positive test results, particularly if test specificity is low.
Single-dose antimicrobial chemotherapy at time of departure^c^	Conventional antimicrobial agents [9,30]	US$0.03–US$0.12 (Doxycycline); US$0.08–US$0.40 (Ciprofloxacin) [53]	High compliance can be expected with a single-dose regimen
	Azithromycin	US$0.52–US$1.32 [53]	
Early-initiated antimicrobial chemotherapy beginning 1 wk prior to departure^d^	3-d course with conventional antimicrobial agents	US$0.24–US$0.48 [53]	Lower treatment compliance may be expected if peacekeepers are required to take therapies during their leave period.
Oral cholera vaccine	Killed bivalent whole-cell vaccine without B subunit (Shanchol, Shantha Biotechnics, Hyderabad, India)	US$3.70 [54]	Direct costs do not include those associated with cold chain shipment and storage infrastructure. Vaccine must be administered 5 wk before departure (Fig 2). Vaccine supplies are limited; the number of doses required to immunize at-risk peacekeeping forces represents roughly 16% of global production capacity for either vaccine as of 2012 [54].
	Killed bivalent whole-cell vaccine with B subunit (Dukoral, Janssen, Inc., Toronto, Canada)	US$9.40–US$18.80 [54]	

^a^Costs are presented in current US dollars.

^b^Ranges represent lower bound–upper bound based on reported costs in the cited sources.

^c^Single-dose antimicrobial regimens consider 300 mg of doxycycline (WHO recommendation [8]) and 1,000 mg of ciprofloxacin.

^d^Early-initiated antimicrobial chemoprophylaxis consider 500 mg of tetracycline administered four times daily for 3 d (WHO recommendation [8]).

Vaccine availability also presents a limitation to implementing OCV interventions. Of 104,928 active-duty UN peacekeeping personnel in 2015, 74% were deployed from WHO-classified cholera-endemic countries. Considering most deployments are 6 mo long, over 310,000 OCV doses would be required to immunize peacekeepers departing endemic countries each year, representing 16% of total annual production capacity for either Shanchol or Dukoral (2 million doses each as of 2012 [54]).

## Discussion

The cholera outbreak in Haiti arose from a confluence of preventable circumstances. Systemic inadequacies in sanitation infrastructure made Haiti vulnerable to water-borne disease [60], like other disaster-affected settings where peacekeeping operations are undertaken. Mass personnel movements from a cholera-endemic country and deficient waste management practices at a MINUSTAH base led to the introduction of *V*. *cholerae* to a susceptible population. Prior to the outbreak, there were no biomedical interventions in place to prevent its occurrence despite the recognized risk for spread of infectious diseases from military to civilian populations [61]. While the UN has been reluctant to implement interventions in the wake of the epidemic in part due to uncertainties surrounding their effectiveness [12,13], our findings suggest antimicrobial chemoprophylaxis reduces the risk of disease introduction by over 90%. The low costs and minimal logistical burden of chemoprophylaxis relative to the other interventions suggest this approach warrants consideration as a strategy to limit risk for cholera introduction in future peacekeeping operations.

Prospective recommendations for OCV use during peacekeeping deployments had been in place prior to the outbreak [62]. Our analysis suggests antimicrobial chemoprophylaxis of peacekeepers beginning 1 wk prior to deployment is more effective than OCV in preventing epidemic introduction, and is also less expensive and easier to implement. While screening via RDTs offers similar levels of protection in comparison to early-initiated antimicrobial chemoprophylaxis, logistical and cost constraints must be considered in evaluating the two intervention strategies. In contrast to conventional culture, administering RDTs using enriched rectal swab samples is simple to perform outside laboratories and requires minimal personnel time. However, false positive results are expected in large battalions even if the screening procedure has high specificity, suggesting confirmatory diagnoses or provision of antimicrobial drugs may be warranted for individuals with positive test outcomes. This logistical constraint was acknowledged in a follow-up report by the UN [12,13]. Our analysis provides quantitative support for comparing the different interventions in terms of their impact on cholera transmission, and demonstrates that screening and OCV interventions would not surpass early-initiated chemoprophylaxis in effectiveness.

Although effective, prophylactic use of antimicrobial drugs for cholera is controversial [8,9,13]. Intensive population-wide tetracycline prophylaxis has been associated with local emergence of drug resistance [63]. In addition, resistance to tetracycline derivatives is prevalent in *V*. *cholerae* depending on geographical region [64]. Azithromycin is an attractive alternative: a single-dose regimen is efficacious in resolving cholera diarrhea and pathogen excretion [49], and resistance in *V*. *cholerae* is comparatively rare [65]. We found single-dose azithromycin to be an optimal strategy if its superiority for cholera treatment translates to high prophylactic efficacy. Furthermore, targeted chemoprophylaxis among strictly-defined groups such as peacekeeping personnel imposes low or negligible selective pressure for drug resistance relative to historical population-based approaches, and has been advocated as a cholera control strategy [19,60]. To date, chemoprophylaxis studies have used differing drug classes and dosing, and have measured bacteriological outcomes inconsistently [9]. Trials are needed to define optimal antimicrobial regimens for cholera chemoprophylaxis. In view of prevalent resistance to conventional therapies, assessing the prophylactic efficacy of azithromycin is of particular priority.

It is uncertain how many infected peacekeepers introduced cholera to Haiti. Considering a wide range of possible incidence rates in Nepal, we identified statistical support for the hypothesis that one infected peacekeeper initiated the outbreak (S3 Table), consistent with genomic analyses supporting a single-source introduction [5,66,67]. There is no record of any peacekeeper experiencing symptoms in Haiti or before departure, and *V*. *cholerae* was not detected in personnel or camp sewage in October 2010 [6,7]. These factors suggest secondary generations of infection did not occur among peacekeepers. The high estimated *R*_0_ for river-mediated transmission, underlying the observed incidence of over 2,000 symptomatic cases per day nationwide by 22 October [6], distinguishes the Artibonite watershed as a high-risk environment for cholera introduction [25,60].

Previously-reported transmission models have not accounted for the dose-response relationship between *V*. *cholerae* exposure and the probability of symptoms in addition to infection [38]. Our model links dose-response observations from human challenge experiments to temporal variability in the distribution of symptomatic and asymptomatic infections [23]. This approach provides a novel explanation for the explosive nature of cholera epidemics such as the Haiti outbreak, and addresses previous uncertainty [6] as to how a small number of asymptomatic peacekeepers could instigate a large outbreak despite low initial levels of *V*. *cholerae* contamination. A quiescent initial increase in asymptomatic cases, which result from lower infectious doses, precedes the precipitous rise in observed cases in our model after a threshold level of environmental contamination is reached.

We consider how the interventions affect transmission from asymptomatic peacekeepers under the assumption that existing protocols for isolation and clinical management of diarrheal cases are effective in preventing symptomatic peacekeepers from transmitting. Because deficient sanitation infrastructure at peacekeeping bases could undermine the effectiveness of existing protocols, proper sewage disposal must be implemented in tandem with biomedical interventions to limit risk for cholera introduction.

Our analysis is subject to several limitations. First, external factors affecting *V*. *cholerae* concentration in the environment mediate the relationship between bacterial shedding and transmission risk [68], limiting our ability to infer how lower levels of *V*. *cholerae* shedding among asymptomatic OCV recipients impact the relative transmission risk of immunized and non-immunized asymptomatic carriers. Thus, our finding that the effectiveness of OCV varies widely depending on the relationship between reduced bacterial shedding and transmission risk undermines support for OCV as a preventive strategy (S8 Table). Our analysis did not consider single-dose OCV administration [69,70], which may be more logistically feasible than two-dose schedules given costs and limited vaccine stockpiles, or in the event that peacekeepers are deployed on short notice during acute crises. In addition, evidence that the vaccine does not confer direct protection against infection comes from limited studies; one recruited healthy subjects with no previous cholera exposure [11], and the other reported incomplete details on follow-up of asymptomatic individuals [10]. We found that OCV would deliver comparable effectiveness to prophylaxis only if vaccines prevent 50% of infections in addition to reducing bacterial shedding among recipients experiencing infection. Protection against infection therefore merits attention in studies of next-generation cholera vaccines including CVD 103-HgR. Unlike antimicrobials that reduce overall risk of infection, OCV could marginally increase peacekeepers’ risk of unknowingly importing *V*. *cholerae* by increasing the fraction of cases that are asymptomatic.

Last, the UN has previously acknowledged that commercial RDTs have only been field-validated among symptomatic cholera cases [12,13]. Validation of RDTs for detecting *V*. *cholerae* in enriched specimens from asymptomatic carriers is a research priority, as this approach could greatly expand low-cost options for screening individuals at risk of transmission. Together with cost and logistical considerations, uncertainty regarding the effectiveness of OCV and screening interventions strengthens the case for chemoprophylaxis as the preferred preventive strategy.

While the role of large-scale international personnel deployments in the global spread of cholera and other infectious diseases is not known, exposure to infectious disease agents in low-income countries that deploy and receive the majority of peacekeeping forces has motivated previous calls for enhanced infectious disease surveillance among troops [61,71]. Whereas the previous absence of cholera from Haiti contributed to interest in ascertaining the geographic origin of the 2010 epidemic [4], importation events affecting peacekeeper-receiving countries where sporadic, epidemic, or endemic cholera transmission already occurs may be less readily detected.

Inadequate water and sanitation facilities and the lack of population immunity to cholera in Haiti put this country at exceptionally high risk for an epidemic following contamination of the Artibonite watershed. However, UN peacekeeping missions serve populations affected by geopolitical and humanitarian emergencies whose vulnerable living circumstances bring about high risk for cholera transmission [60,72,73]. Although our model primarily offers a retrospective analysis of how several interventions might have led to different outcomes in Haiti, inferences about the comparative effectiveness of the interventions are generalizable to other settings. In particular, estimated reductions in importation probability through screening and prophylaxis hold for peacekeepers departing any country with endemic cholera transmission. In this respect, we identify that these interventions offer at minimum 81% and 89% effectiveness, respectively, against asymptomatic *V*. *cholerae* importation during future global deployments. As of 2015, over 75,000 UN peacekeeping personnel actively serving at international missions were deployed from WHO-classified cholera-endemic countries [1,74]. With operations at this scale, the risk for further importation events is high under existing protocols even if individual peacekeepers or battalions have a low probability of carrying *V*. *cholerae*.

Troop-deploying countries are currently encouraged to provide OCVs to protect peacekeepers against cholera during deployments to cholera-endemic countries [7,12,13]. Our finding that biomedical interventions serve the dual purpose of reducing risk for cholera introduction in troop-receiving countries suggests existing UN protocols are suboptimal. Chemoprophylaxis with antimicrobial drugs, targeted judiciously to peacekeepers deployed from cholera-endemic settings, offers superior protection against cholera introduction relative to immunization and imposes lower costs and logistical burden than RDT-based screening or immunization with OCV. Combined interventions utilizing OCV with chemoprophylaxis offer particularly high effectiveness. Quantitative assessments provide support and guidance for policymakers weighing the merits and cost-sharing of different interventions.

## Supporting Information

S1 Table Symptom probability in endemic Asian settings. (PDF)

S2 Table Prevalence of asymptomatic infection for differing endemic cholera incidence rates. (PDF)

S3 Table Status quo importation probabilities by number of infected arrivals. (PDF)

S4 Table Sensitivity analysis: reductions in case probability with varying RDT performance. (PDF)

S5 Table Sensitivity analysis: false positive tests expected with varying RDT specificity. (PDF)

S6 Table Sensitivity analysis: case probabilities at reduced antimicrobial efficacy. (PDF)

S7 Table Sensitivity analysis: outcome probabilities with varying vaccine efficacy against transmission. (PDF)

S8 Table Sensitivity analysis: case probabilities using single-dose azithromycin at time of departure. (PDF)

S9 Table Sensitivity analysis: case probabilities using single-dose azithromycin at time of departure combined with oral cholera vaccine. (PDF)

S10 Table Sensitivity analysis: combined intervention effectiveness at lower vaccine efficacy against transmission and reduced antimicrobial efficacy—time-of-departure administration. (PDF)

S11 Table Sensitivity analysis: combined intervention effectiveness at lower vaccine efficacy against transmission and reduced antimicrobial efficacy—early-course administration. (PDF)

S12 Table Sensitivity analysis: combined intervention effectiveness at lower vaccine efficacy against transmission using single-dose azithromycin. (PDF)

S13 Table Sensitivity analysis: basic reproductive numbers (*R*_0_) with varying relative infectiousness of symptomatic cases. (PDF)

S14 Table Sensitivity analysis: case probabilities with varying relative infectiousness of symptomatic cases in local transmission. (PDF)

S15 Table Sensitivity analysis: fitted parameters with varying relative infectiousness of symptomatic cases. (PDF)

S16 Table Sensitivity analysis: case probabilities with varying vaccine protection against infection. (PDF)

S1 Text Supplemental methods. (PDF)

## Abbreviations

ACP: Antimicrobial chemoprophylaxis
CrI: credible interval
MINUSTAH: United Nations Peacekeeping Mission in Haiti
OCVs: oral cholera vaccines
PYAR: person-years at risk
RDT: rapid diagnostic test
UN: United Nations
