# Supplementary material for: Strategies to Prevent Cholera Introduction during International Personnel Deployments: A Computational Modeling Analysis Based on the 2010 Haiti Outbreak
Source: PLoS Med. 2016 Jan 26;13(1):e1001947. doi: 10.1371/journal.pmed.1001947 (PMC4727895; doi:10.1371/journal.pmed.1001947)
Supplement: S1 Table — (PDF) [file pmed.1001947.s001.pdf]

## S1 Table. Symptom probability in endemic Asian settings.

| Setting | Citation | Asymptomatic, culture-positive ($\beta$) | Symptomatic, culture-positive ($\alpha$) | Symptom probability (%)[b] |
|---|---|---|---|---|
| Bangladesh | [14] | 23 | 33 | 58.9 (45.9, 71.3) |
| Pakistan | [15][a] | 35 | 16 | 31.4 (19.5, 44.6) |
| Bangladesh | [16] | 22 | 8 | 26.7 (12.7, 43.5) |
| Philippines | [17] | 48 | 7 | 12.7 (5.4, 22.6) |
| Bangladesh | [18] | 404 | 161 | 28.5 (24.9, 32.3) |
| Bangladesh | [19] | 239 | 29 | 10.8 (7.4, 14.8) |
| **Pooled est.** | | | | **24.2 (14.4, 40.7)** |

[a]We limited the data from the Bart et al. study to contacts of index patients shedding the El Tor biotype.
[b]Estimates from individual studies are presented with 95% quantiles following the Beta distribution, whereas the 95% interval for the pooled estimate results from the inverse variance weighted random effects model.
