# Supplementary material for: Strategies to Prevent Cholera Introduction during International Personnel Deployments: A Computational Modeling Analysis Based on the 2010 Haiti Outbreak
Source: PLoS Med. 2016 Jan 26;13(1):e1001947. doi: 10.1371/journal.pmed.1001947 (PMC4727895; doi:10.1371/journal.pmed.1001947)
Supplement: S2 Table — (PDF) [file pmed.1001947.s002.pdf]

S2 Table. Prevalence of asymptomatic infection for differing endemic cholera incidence rates.

Scenario	Incidence rate	Prevalence (per 100,000 peacekeepers) Time of departure	Time of arrival
Status quo	0.5/1000 PYAR	2.9 (1.3, 5.5)	1.8 (0.8, 3.4)
	1.0/1000 PYAR	5.7 (2.6, 11.1)	3.5 (1.6, 6.8)
	2.0/1000 PYAR	11.5 (5.2, 22.1)	7.1 (3.2, 13.6)
	5.0/1000 PYAR	28.7 (13.1, 55.3)	17.7 (8.1, 34.1)
	10.0/1000 PYAR	57.4 (26.2, 110.6)	35.4 (16.2, 68.2)
RDT screening[b]	0.5/1000 PYAR		0.3 (0.1, 0.7)
	1.0/1000 PYAR		0.6 (0.3, 1.3)
	2.0/1000 PYAR		1.3 (0.6, 2.6)
	5.0/1000 PYAR		3.2 (1.5, 6.5)
	10.0/1000 PYAR		6.5 (2.9, 13.1)
Time-of-departure prophylaxis[b]	0.5/1000 PYAR		1.0 (0.4, 2.3)
	1.0/1000 PYAR		2.2 (0.9, 4.6)
	2.0/1000 PYAR		4.3 (1.8, 9.1)
	5.0/1000 PYAR		10.8 (4.4, 22.8)
	10.0/1000 PYAR		21.5 (8.8, 45.5)
Early-initiated prophylaxis	0.5/1000 PYAR	0.6 (0.2, 1.6)	0.2 (0.1, 0.6)
	1.0/1000 PYAR	1.2 (0.4, 3.1)	0.4 (0.1, 1.3)
	2.0/1000 PYAR	2.3 (0.8, 6.2)	0.8 (0.2, 2.5)
	5.0/1000 PYAR	5.8 (2.0, 15.5)	1.9 (0.6, 2.3)
	10.0/1000 PYAR	11.6 (4.0, 31.1)	3.9 (1.1, 12.6)

PYAR: person-years at risk (incidence rate denominator).

[a]Prevalence is reported as median (95% CrI) as inferred via Monte Carlo realizations of the distributions presented in S1 Text (§1).

[b]Time-of-departure prevalence is equal to the status quo expectation for RDT screening and time-of-departure prophylaxis.
