# Supplementary material for: Strategies to Prevent Cholera Introduction during International Personnel Deployments: A Computational Modeling Analysis Based on the 2010 Haiti Outbreak
Source: PLoS Med. 2016 Jan 26;13(1):e1001947. doi: 10.1371/journal.pmed.1001947 (PMC4727895; doi:10.1371/journal.pmed.1001947)
Supplement: S3 Table — (PDF) [file pmed.1001947.s003.pdf]

S3 Table. Status quo importation probabilities by number of infected arrivals.

Incidence rate	Any infected arrival	1 infected arrival		2 infected arrivals		3 infected arrivals		More than 3 infected arrivals
	Absolute (%)[a]	Absolute (%)[a]	*w* (%)[a]	Absolute (%)[a]	*w* (%)[a]	Absolute (%)[a]	*w* (%)[a]	Absolute (%)[a]
0.5/1000 PYAR	0.8 (0.4, 1.6)	0.8 (0.4, 1.5)	99.6 (99.2, 99.8)	$3.2e^{-3}$ ($6.5e^{-4}$, $1.2e^{-2}$)	0.4 (0.2, 0.8)	$8.5e^{-6}$ ($7.8e^{-7}$, $6.2e^{-5}$)	$1.1e^{-3}$ ($2.2e^{-4}$, $4.0e^{-3}$)	$1.7e^{-8}$ ($7.0e^{-10}$, $2.4e^{-7}$)
1.0/1000 PYAR	1.6 (0.7, 3.1)	1.6 (0.8, 3.0)	99.2 (98.4, 99.6)	$1.3e^{-2}$ ($2.6e^{-3}$, $4.7e^{-2}$)	0.8 (0.4, 1.5)	$6.8e^{-5}$ ($6.2e^{-6}$, $4.9e^{-4}$)	$0.4e^{-3}$ ($8.6e^{-4}$, $1.6e^{-2}$)	$2.7e^{-7}$ ($1.1e^{-8}$, $3.8e^{-6}$)
2.0/1000 PYAR	3.2 (1.4, 6.1)	3.1 (1.4, 5.9)	98.4 (96.9, 99.3)	$5.0e^{-2}$ ($1.0e^{-2}$, $1.8e^{-1}$)	1.6 (0.7, 3.0)	$5.3e^{-4}$ ($4.9e^{-5}$, $3.8e^{-3}$)	$1.7e^{-2}$ ($3.4e^{-3}$, $6.3e^{-2}$)	$4.3e^{-6}$ ($1.8e^{-7}$, $6.0e^{-5}$)
5.0/1000 PYAR	7.7 (3.6, 14.5)	7.4 (3.5, 13.4)	96.0 (92.4, 98.2)	0.3 (0.1–1.0)	3.9 (1.8, 7.2)	$7.9e^{-3}$ ($7.6e^{-4}$, $5.4e^{-2}$)	0.1 (0.0, 0.4)	$1.6e^{-4}$ ($6.8e^{-6}$, $2.2e^{-3}$)
10.0/1000 PYAR	14.8 (7.0, 26.9)	13.7 (6.7, 22.9)	92.2 (85.3, 96.4)	1.1 (0.2–3.6)	7.4 (3.5, 13.3)	0.1 (0.0, 0.4)	0.4 (0.1, 1.4)	$2.4e^{-3}$ ($1.1e^{-4}$, $3.1e^{-2}$)

PYAR: person-years at risk (incidence rate denominator).

[a]Notation: $3.2e^{-3}$ = $(3.2)10^{-3}$. Probabilities are presented as median (95% CrI) as inferred from the Binomial distributions inputting status quo importation probabilities from S3 Table.
