# Supplementary material for: Strategies to Prevent Cholera Introduction during International Personnel Deployments: A Computational Modeling Analysis Based on the 2010 Haiti Outbreak
Source: PLoS Med. 2016 Jan 26;13(1):e1001947. doi: 10.1371/journal.pmed.1001947 (PMC4727895; doi:10.1371/journal.pmed.1001947)
Supplement: S4 Table — (PDF) [file pmed.1001947.s004.pdf]

S4 Table. Sensitivity analysis: reductions in case probability with varying RDT performance.

Background cholera incidence rate	Reduction in importation/case probability (%)[a,b] Sensitivity, specificity						
	99%, 99%	95%, 95%	90%, 90%	80%, 80%	70%, 70%	60%, 60%	50%, 50%
0.5/1000 PYAR	83.2	80.5	77.1	70.3	63.5	56.7	49.9
1.0/1000 PYAR	83.1	80.4	77.0	70.2	63.4	56.6	49.8
2.0/1000 PYAR	83.0	80.3	76.9	70.1	63.3	56.5	49.7
5.0/1000 PYAR	82.8	80.0	76.5	69.6	62.8	56.0	49.2
10.0/1000 PYAR	82.3	79.5	75.9	68.9	62.0	55.1	48.3

PYAR: person-years at risk (incidence rate denominator).
[a]Values reported in table are the proportional reduction in the probability of any infected peacekeeper for sensitivity and specificity inputs in the formula presented in S1 Text §2.2.
[b]Reductions in importation probability are equivalent to reductions in case probability for screening interventions.
