# Supplementary material for: Strategies to Prevent Cholera Introduction during International Personnel Deployments: A Computational Modeling Analysis Based on the 2010 Haiti Outbreak
Source: PLoS Med. 2016 Jan 26;13(1):e1001947. doi: 10.1371/journal.pmed.1001947 (PMC4727895; doi:10.1371/journal.pmed.1001947)
Supplement: S5 Table — (PDF) [file pmed.1001947.s005.pdf]

S5 Table. Sensitivity analysis: false positive tests expected with varying RDT specificity.

Specificity	Peacekeepers barred from deployment due to false positive test, *n* of 454
99%	4 (1, 9)
95%	23 (14, 32)
90%	45 (33, 58)
80%	91 (74, 108)
70%	136 (117, 156)
60%	182 (161, 202)
50%	227 (206, 248)

Values are reported as median (95% CrI) based on quantiles of the corresponding Binomial distributions (S1 Text §2.2). Expected false positive outcomes did not differ across the range of incidence rates assessed (0.5/1000 PYAR to 10.0/1000 PYAR).
