# Supplementary material for: Strategies to Prevent Cholera Introduction during International Personnel Deployments: A Computational Modeling Analysis Based on the 2010 Haiti Outbreak
Source: PLoS Med. 2016 Jan 26;13(1):e1001947. doi: 10.1371/journal.pmed.1001947 (PMC4727895; doi:10.1371/journal.pmed.1001947)
Supplement: S6 Table — (PDF) [file pmed.1001947.s006.pdf]

S6 Table. Sensitivity analysis: case probabilities at reduced antimicrobial efficacy.

	Background cholera incidence rate	Time-of-departure		Early-initiated	
		Probability (%)[a,b]	Effectiveness (%)[a,b]	Probability (%)[a,b]	Effectiveness (%)[a,b]
10% efficacy reduction	0.5/1000 PYAR	0.4 (0.2, 0.7)	44.1 (37.2, 49.9)	0.1 (0.0, 0.2)	88.1 (72.4, 94.4)
	1.0/1000 PYAR	0.7 (0.3, 1.5)	44.0 (37.1, 49.8)	0.2 (0.1, 0.5)	88.0 (72.3, 94.4)
	2.0/1000 PYAR	1.5 (0.6, 2.9)	43.8 (36.9, 49.7)	0.3 (0.1, 0.9)	87.9 (72.1, 94.4)
	5.0/1000 PYAR	3.6 (1.6, 7.1)	43.3 (36.3, 49.3)	0.8 (0.3, 2.3)	87.7 (71.7, 94.2)
	10.0/1000 PYAR	7.1 (3.1, 13.8)	42.4 (35.2, 48.7)	1.6 (0.5, 4.6)	87.3 (71.0, 94.1)
25% efficacy reduction	0.5/1000 PYAR	0.4 (0.2, 0.8)	35.8 (28.7, 40.7)	0.1 (0.0, 0.3)	82.4 (60.8, 91.6)
	1.0/1000 PYAR	0.8 (0.4, 1.7)	35.7 (28.6, 40.6)	0.2 (0.1, 0.7)	82.4 (60.7, 91.5)
	2.0/1000 PYAR	1.7 (0.8, 3.3)	35.6 (28.5, 40.5)	0.5 (0.2, 1.3)	82.3 (60.5, 91.5)
	5.0/1000 PYAR	4.1 (1.9, 8.1)	35.1 (28.0, 40.1)	1.1 (0.4, 3.3)	82.0 (59.9, 91.4)
	10.0/1000 PYAR	8.1 (3.7,15.5)	34.2 (26.9, 39.5)	2.3 (0.8, 6.5)	81.4 (58.9, 91.1)
50% efficacy reduction	0.5/1000 PYAR	0.5 (0.2, 1.0)	22.7 (14.7, 27.4)	0.2 (0.1, 0.5)	69.8 (34.7, 85.2)
	1.0/1000 PYAR	1.0 (0.5, 2.0)	22.7 (14.7, 27.3)	0.4 (0.1, 1.1)	69.8 (34.7, 85.2)
	2.0/1000 PYAR	2.0 (0.9, 3.9)	22.6 (14.6, 27.2)	0.8 (0.3, 2.2)	69.6 (34.5, 85.1)
	5.0/1000 PYAR	5.0 (2.2, 9.6)	22.2 (14.4, 26.9)	2.0 (0.7, 5.4)	69.2 (34.0, 84.8)
	10.0/1000 PYAR	9.7 (4.5, 18.2)	21.6 (13.9, 26.3)	3.9 (1.4, 10.5)	68.5 (33.1, 84.4)

PYAR: person-years at risk (incidence rate denominator).

[a]Case probabilities refer to the likelihood that at least one symptomatic cholera case occurs in the community. Effectiveness is defined as the reduction in this probability relative to its estimate under status quo protocols.

[b]Estimates are reported as median (95% CrI), as obtained via bootstrap resampling.
