# Supplementary material for: Strategies to Prevent Cholera Introduction during International Personnel Deployments: A Computational Modeling Analysis Based on the 2010 Haiti Outbreak
Source: PLoS Med. 2016 Jan 26;13(1):e1001947. doi: 10.1371/journal.pmed.1001947 (PMC4727895; doi:10.1371/journal.pmed.1001947)
Supplement: S7 Table — (PDF) [file pmed.1001947.s007.pdf]

S7 Table. Sensitivity analysis: outcome probabilities with varying vaccine efficacy against transmission.

Relative shedding rate[a]	Background cholera incidence rate	Probability[b,c] (%)	Effectiveness[c,d] (%)
ϕ = 0.0097	0.5/1000 PYAR	0.2 (0.1, 0.3)	75.3 (73.9, 76.7)
	1.0/1000 PYAR	0.3 (0.1, 0.6)	75.2 (73.8, 76.6)
	2.0/1000 PYAR	0.6 (0.3, 1.2)	75.2 (73.8, 76.5)
	5.0/1000 PYAR	1.6 (0.7, 3.0)	74.9 (73.5, 76.3)
	10.0/1000 PYAR	3.1 (1.4, 5.9)	74.5 (73.2, 76.0)
ϕ = 0.0194	0.5/1000 PYAR	0.3 (0.1, 0.5)	60.9 (59.3, 62.6)
	1.0/1000 PYAR	0.5 (0.2, 1.0)	60.9 (59.2, 62.5)
	2.0/1000 PYAR	1.0 (0.5, 2.0)	60.7 (59.1, 62.4)
	5.0/1000 PYAR	2.5 (1.2, 4.8)	60.4 (58.7, 62.0)
	10.0/1000 PYAR	5.0 (2.3, 9.2)	59.7 (58.0, 61.5)
ϕ = 0.0388	0.5/1000 PYAR	0.4 (0.2, 0.7)	44.3 (42.5, 46.1)
	1.0/1000 PYAR	0.7 (0.3, 1.4)	44.2 (42.4, 46.0)
	2.0/1000 PYAR	1.5 (0.7, 2.8)	44.1 (42.3, 45.9)
	5.0/1000 PYAR	3.6 (1.7, 6.8)	43.7 (41.9, 45.4)
	10.0/1000 PYAR	7.0 (3.3, 13.1)	43.0 (41.1, 44.9)
ϕ = 0.0776	0.5/1000 PYAR	0.5 (0.2, 0.9)	27.4 (25.5, 29.3)
	1.0/1000 PYAR	1.0 (0.4, 1.8)	27.4 (25.4, 29.2)
	2.0/1000 PYAR	1.9 (0.9, 3.6)	27.2 (25.3, 29.1)
	5.0/1000 PYAR	4.7 (2.2, 8.8)	26.8 (24.9, 28.7)
	10.0/1000 PYAR	9.1 (4.3, 16.8)	26.2 (24.2, 28.1)
ϕ = 0.194	0.5/1000 PYAR	0.6 (0.3, 1.1)	10.9 (8.9, 12.7)
	1.0/1000 PYAR	1.2 (0.5, 2.2)	10.8 (8.9, 12.6)
	2.0/1000 PYAR	2.3 (1.1, 4.4)	10.8 (8.9, 12.6)
	5.0/1000 PYAR	5.7 (2.7, 10.7)	10.6 (8.8, 12.4)
	10.0/1000 PYAR	11.1 (5.2, 20.2)	10.3 (8.5, 12.0)
ϕ = 0.388	0.5/1000 PYAR	0.6 (0.3, 1.2)	4.9 (3.0, 6.7)
	1.0/1000 PYAR	1.2 (0.6, 2.4)	4.9 (3.0, 6.7)
	2.0/1000 PYAR	2.5 (1.1, 4.7)	4.8 (3.0, 6.7)
	5.0/1000 PYAR	6.1 (2.8, 11.4)	4.7 (2.9, 6.5)
	10.0/1000 PYAR	11.8 (5.6, 21.4)	4.5 (2.8, 6.2)
ϕ = 0.5	0.5/1000 PYAR	0.6 (0.3, 1.2)	3.5 (1.7, 5.4)
	1.0/1000 PYAR	1.3 (0.6, 2.4)	3.5 (1.7, 5.3)
	2.0/1000 PYAR	2.5 (1.2, 4.8)	3.5 (1.7, 5.3)
	5.0/1000 PYAR	6.2 (2.9, 11.6)	3.4 (1.7, 5.1)
	10.0/1000 PYAR	12.0 (5.6, 21.8)	3.2 (1.6, 4.9)

PYAR: Person-years at risk (incidence rate denominator).

[a]Relative shedding is quantified as *V. cholerae* output among vaccinated (relative to unvaccinated) asymptomatic subjects. Based on a relative concentration of 0.0194 *V. cholerae* colony-forming units per milliliter of stool in [11], we consider reductions of 0.0097 (half the reported ratio) to 0.388 (20 times the reported ratio of shedding) and 0.5 (26 times the reported ratio).

[b]We report probability for a case to occur in Haiti based on the differing background incidence rate estimates.

[c]Estimates are reported as median (95% CrI) based on bootstrap resampling.

[d]Effectiveness is measured as the reduction in probability that a case occurs in Haiti.
