# Supplementary material for: Strategies to Prevent Cholera Introduction during International Personnel Deployments: A Computational Modeling Analysis Based on the 2010 Haiti Outbreak
Source: PLoS Med. 2016 Jan 26;13(1):e1001947. doi: 10.1371/journal.pmed.1001947 (PMC4727895; doi:10.1371/journal.pmed.1001947)
Supplement: S9 Table — (PDF) [file pmed.1001947.s009.pdf]

S9 Table. Sensitivity analysis: case probabilities using single-dose azithromycin at time of departure combined with oral cholera vaccine.

Background cholera incidence rate	10% superior efficacy		25% superior efficacy		50% superior efficacy	
	Probability (%)	Effectiveness (%)	Probability (%)	Effectiveness (%)	Probability (%)	Effectiveness (%)
0.5/1000 PYAR	0.07 (0.03, 0.15)	89.3 (87.3, 91.2)	0.04 (0.02, 0.10)	93.4 (91.6, 95.3)	0.014 (0.003, 0.041)	97.9 (96.2, 99.3)
1.0/1000 PYAR	0.14 (0.06, 0.29)	89.3 (87.3, 91.2)	0.09 (0.03, 0.19)	93.3 (91.6, 95.3)	0.027 (0.006, 0.082)	97.8 (96.2, 99.3)
2.0/1000 PYAR	0.3 (0.1, 0.6)	89.2 (87.2, 91.2)	0.2 (0.1, 0.4)	93.3 (91.5, 95.3)	0.06 (0.01, 0.16)	97.8 (96.1, 99.3)
5.0/1000 PYAR	0.7 (0.3, 1.4)	89.0 (86.9, 91.1)	0.4 (0.2, 1.0)	93.2 (91.3, 95.2)	0.14 (0.03, 0.41)	97.8 (96.0, 99.2)
10.0/1000 PYAR	1.4 (0.6, 2.8)	88.8 (86.5, 90.9)	0.9 (0.3, 1.9)	93.0 (90.9, 95.1)	0.3 (0.1, 0.8)	97.7 (95.9, 99.2)

PYAR: person-years at risk (incidence rate denominator).

[a]Case probabilities refer to the likelihood that at least one symptomatic cholera case occurs in the community. Effectiveness is defined as the reduction in this probability relative to its estimate under status quo protocols.

[b]Estimates are reported as median (95% CrI), as obtained via bootstrap resampling.
