# Supplementary material for: Strategies to Prevent Cholera Introduction during International Personnel Deployments: A Computational Modeling Analysis Based on the 2010 Haiti Outbreak
Source: PLoS Med. 2016 Jan 26;13(1):e1001947. doi: 10.1371/journal.pmed.1001947 (PMC4727895; doi:10.1371/journal.pmed.1001947)
Supplement: S11 Table — (PDF) [file pmed.1001947.s011.pdf]

S11 Table. Sensitivity analysis: Combined intervention effectiveness at lower vaccine efficacy against transmission and reduced antimicrobial efficacy, early-course administration.

Vaccine efficacy against transmission	Background cholera incidence rate	10% reduced antimicrobial efficacy		25% reduced antimicrobial efficacy		50% reduced antimicrobial efficacy	
		Case probability (%)[a,b]	Effectiveness (%)[a,b]	Case probability (%)[a,b]	Effectiveness (%)[a,b]	Case probability (%)[a,b]	Effectiveness (%)[a,b]
ϕ = 0.0388	0.5/1000 PYAR	0.04 (0.01, 0.1)	94.7 (87.7, 97.5)	0.1 (0.0, 0.2)	91.6 (81.3, 96.0)	0.1 (0.0, 0.3)	85.4 (68.3, 92.8)
	1.0/1000 PYAR	0.1 (0.0, 0.2)	94.7 (87.6, 97.5)	0.1 (0.0, 0.3)	91.6 (81.3, 96.0)	0.2 (0.1, 0.5)	85.4 (68.2, 92.8)
	2.0/1000 PYAR	0.1 (0.0, 0.4)	94.6 (87.6, 97.5)	0.2 (0.1, 0.6)	91.5 (81.2, 96.0)	0.4 (0.1, 1.1)	85.3 (68.1, 92.7)
	5.0/1000 PYAR	0.3 (0.1, 1.0)	94.5 (87.4, 97.4)	0.5 (0.2, 1.6)	91.4 (80.8, 95.9)	0.9 (0.3, 2.7)	85.0 (67.7, 92.6)
	10.0/1000 PYAR	0.7 (0.2, 2.1)	94.4 (87.0, 97.4)	1.1 (0.4, 3.1)	91.1 (80.2, 95.7)	1.9 (0.7, 5.2)	84.6 (66.8, 92.4)
ϕ = 0.0776	0.5/1000 PYAR	0.05 (0.02, 0.2)	92.5 (82.9, 96.5)	0.1 (0.0, 0.2)	89.0 (75.4, 94.7)	0.1 (0.0, 0.4)	79.4 (55.3, 89.9)
	1.0/1000 PYAR	0.1 (0.0, 0.3)	92.5 (82.9, 96.5)	0.1 (0.0, 0.4)	89.0 (75.3, 94.7)	0.3 (0.1, 0.8)	79.3 (55.1, 89.9)
	2.0/1000 PYAR	0.2 (0.1, 0.6)	92.5 (82.8, 96.5)	0.3 (0.1, 0.8)	88.9 (75.1, 94.7)	0.5 (0.2, 1.5)	79.2 (54.9, 89.8)
	5.0/1000 PYAR	0.5 (0.2, 1.5)	92.3 (82.4, 96.4)	0.7 (0.2, 2.1)	88.7 (74.6, 94.6)	1.3 (0.5, 3.7)	78.9 (54.5, 89.6)
	10.0/1000 PYAR	1.0 (0.3, 2.9)	92.1 (81.8, 96.3)	1.4 (0.5, 4.1)	88.3 (73.9, 94.4)	2.7 (0.9, 7.3)	78.3 (53.6, 89.3)
ϕ = 0.194	0.5/1000 PYAR	0.1 (0.0, 0.2)	90.0 (76.9, 95.3)	0.1 (0.0, 0.3)	84.7 (65.9, 92.7)	0.2 (0.1, 0.5)	74.5 (44.8, 87.5)
	1.0/1000 PYAR	0.1 (0.0, 0.4)	89.9 (76.8, 95.3)	0.2 (0.1, 0.6)	84.7 (65.8, 92.6)	0.3 (0.1, 0.9)	74.4 (44.7, 87.5)
	2.0/1000 PYAR	0.3 (0.1, 0.8)	89.9 (76.7, 95.3)	0.4 (0.1, 1.2)	84.6 (65.6, 92.6)	0.7 (0.2, 1.9)	74.3 (44.5, 87.4)
	5.0/1000 PYAR	0.7 (0.2, 2.0)	89.7 (76.3, 95.2)	1.0 (0.3, 2.9)	84.3 (65.1, 92.5)	1.7 (0.6, 4.6)	73.8 (44.0, 87.2)
	10.0/1000 PYAR	1.3 (0.4, 3.9)	89.3 (75.6, 95.0)	2.0 (0.7, 5.7)	83.8 (64.3, 92.2)	3.3 (1.2, 9.0)	73.2 (42.9, 86.7)
ϕ = 0.388	0.5/1000 PYAR	0.1 (0.0, 0.2)	88.9 (74.4, 94.8)	0.1 (0.0, 0.3)	83.1 (62.4, 91.9)	0.2 (0.1, 0.5)	71.9 (39.1, 86.2)
	1.0/1000 PYAR	0.1 (0.0, 0.4)	88.8 (74.3, 94.8)	0.2 (0.1, 0.6)	83.1 (62.3, 91.9)	0.4 (0.1, 1.0)	71.8 (39.0, 86.2)
	2.0/1000 PYAR	0.3 (0.1, 0.9)	88.8 (74.1, 94.7)	0.4 (0.1, 1.3)	83.0 (62.1, 91.8)	0.7 (0.3, 2.1)	71.7 (38.9, 86.1)
	5.0/1000 PYAR	0.7 (0.2, 2.2)	88.6 (73.7, 94.6)	1.1 (0.4, 3.2)	82.7 (61.5, 91.7)	1.8 (0.6, 5.0)	71.2 (38.4, 85.8)
	10.0/1000 PYAR	1.4 (0.5, 4.3)	88.2 (73.0, 94.5)	2.2 (0.7, 6.2)	82.2 (60.5, 91.4)	3.6 (1.3, 9.8)	70.5 (37.5, 85.5)
ϕ = 0.5	0.5/1000 PYAR	0.1 (0.0, 0.2)	88.7 (73.8, 94.7)	0.1 (0.0, 0.3)	83.1 (62.2, 91.9)	0.2 (0.1, 0.5)	70.6 (36.1, 85.5)
	1.0/1000 PYAR	0.1 (0.0, 0.4)	88.6 (73.8, 94.7)	0.2 (0.1, 0.6)	83.0 (62.1, 91.9)	0.4 (0.1, 1.1)	70.5 (36.0, 85.5)
	2.0/1000 PYAR	0.3 (0.1, 0.9)	88.6 (73.6, 94.7)	0.4 (0.1, 1.3)	82.9 (62.0, 91.8)	0.8 (0.3, 2.1)	70.4 (35.8, 85.4)
	5.0/1000 PYAR	0.7 (0.2, 2.2)	88.4 (73.2, 94.6)	1.1 (0.4, 3.2)	82.6 (61.4, 91.7)	1.9 (0.7, 5.3)	69.9 (35.3, 85.1)
	10.0/1000 PYAR	1.5 (0.5, 4.4)	88.0 (72.5, 94.4)	2.2 (0.7, 6.3)	82.1 (60.4, 91.4)	3.8 (1.3, 10.3)	69.2 (34.4, 84.7)

PYAR: person-years at risk (incidence rate denominator).

[a]Case probabilities refer to the likelihood that at least one symptomatic cholera case occurs in the community. Effectiveness is defined as the reduction in this probability relative to its estimate under status quo protocols.

[b]Estimates are reported as median (95% CrI), as obtained via bootstrap resampling.
