# Supplementary material for: Strategies to Prevent Cholera Introduction during International Personnel Deployments: A Computational Modeling Analysis Based on the 2010 Haiti Outbreak
Source: PLoS Med. 2016 Jan 26;13(1):e1001947. doi: 10.1371/journal.pmed.1001947 (PMC4727895; doi:10.1371/journal.pmed.1001947)
Supplement: S12 Table — (PDF) [file pmed.1001947.s012.pdf]

S12 Table. Sensitivity analysis: Combined intervention effectiveness at lower vaccine efficacy against transmission using single-dose azithromycin.

Vaccine efficacy against transmission	Background cholera incidence rate	10% super antimicrobial efficacy		25% superior antimicrobial efficacy		50% superior antimicrobial efficacy	
		Case probability (%)[a,b]	Effectiveness (%)[a,b]	Case probability (%)[a,b]	Effectiveness (%)[a,b]	Case probability (%)[a,b]	Effectiveness (%)[a,b]
ϕ = 0.0388	0.5/1000 PYAR	0.1 (0.0, 0.2)	83.2 (80.3, 86.2)	0.1 (0.0, 0.2)	88.2 (85.1, 91.6)	0.02 (0.005, 0.07)	96.5 (93.9, 98.8)
	1.0/1000 PYAR	0.2 (0.1, 0.5)	83.1 (80.2, 86.2)	0.2 (0.1, 0.3)	88.2 (85.1, 91.6)	0.04 (0.009, 0.1)	96.5 (93.9, 98.8)
	2.0/1000 PYAR	0.4 (0.2, 0.9)	83.0 (80.1, 86.1)	0.3 (0.1, 0.7)	88.1 (85.0, 91.5)	0.1 (0.0, 0.3)	96.5 (93.8, 98.8)
	5.0/1000 PYAR	1.1 (0.5, 2.3)	82.8 (79.6, 85.9)	0.8 (0.3, 1.7)	87.9 (84.7, 91.4)	0.2 (0.0, 0.7)	96.4 (93.7, 98.8)
	10.0/1000 PYAR	2.2 (0.9, 4.5)	82.3 (78.8, 85.6)	1.5 (0.6, 3.3)	87.6 (84.1, 91.3)	0.4 (0.1, 1.3)	96.3 (93.3, 98.7)
ϕ = 0.0776	0.5/1000 PYAR	0.2 (0.1, 0.3)	74.9 (70.6, 79.3)	0.1 (0.0, 0.3)	82.5 (78.0, 87.5)	0.03 (0.008, 0.01)	94.2 (89.7, 98.0)
	1.0/1000 PYAR	0.3 (0.1, 0.7)	74.9 (70.5, 79.3)	0.2 (0.1, 0.5)	82.4 (77.9, 87.5)	0.07 (0.02, 0.2)	94.2 (89.7, 98.0)
	2.0/1000 PYAR	0.7 (0.3, 1.4)	74.7 (70.4, 79.2)	0.5 (0.2, 1.0)	82.3 (77.7, 87.4)	0.1 (0.0, 0.4)	94.1 (89.6, 98.0)
	5.0/1000 PYAR	1.6 (0.7, 3.4)	74.4 (69.9, 79.0)	1.1 (0.4, 2.5)	82.1 (77.3, 87.3)	0.4 (0.1, 1.1)	94.0 (89.3, 97.9)
	10.0/1000 PYAR	3.2 (1.3, 6.6)	73.8 (68.9, 78.6)	2.3 (0.9, 4.9)	81.6 (76.4, 87.0)	0.7 (0.2, 2.2)	93.8 (88.8, 98.9)
ϕ = 0.194	0.5/1000 PYAR	0.2 (0.1, 0.5)	65.2 (59.3, 71.2)	0.2 (0.1, 0.4)	74.9 (68.5, 82.1)	0.1 (0.0, 0.2)	90.9 (83.9, 96.9)
	1.0/1000 PYAR	0.5 (0.2, 0.9)	65.1 (59.1, 71.1)	0.3 (0.1, 0.7)	74.8 (68.4, 82.1)	0.1 (0.0, 0.3)	90.9 (83.8, 96.9)
	2.0/1000 PYAR	0.9 (0.4, 1.9)	64.9 (58.9, 71.0)	0.7 (0.2, 1.4)	74.7 (68.2, 82.0)	0.2 (0.0, 0.7)	90.8 (83.7, 96.8)
	5.0/1000 PYAR	2.3 (0.9, 4.6)	64.5 (58.3, 70.8)	1.6 (0.6, 3.5)	74.3 (67.6, 81.8)	0.6 (0.1, 1.7)	90.6 (83.3, 96.8)
	10.0/1000 PYAR	4.5 (1.9, 9.0)	63.7 (57.1, 70.2)	3.2 (1.2, 6.9)	73.7 (66.5, 81.4)	1.2 (0.2, 3.4)	90.3 (82.7, 96.7)
ϕ = 0.388	0.5/1000 PYAR	0.3 (0.1, 0.5)	60.0 (53.3, 66.9)	0.2 (0.1, 0.4)	71.1 (63.8, 79.4)	0.1 (0.0, 0.2)	89.3 (81.2, 96.3)
	1.0/1000 PYAR	0.5 (0.2, 1.1)	59.9 (53.2, 66.9)	0.4 (0.1, 0.8)	71.0 (63.7, 79.4)	0.1 (0.0, 0.4)	89.3 (81.2, 96.3)
	2.0/1000 PYAR	1.0 (0.4, 2.2)	59.8 (52.9, 66.8)	0.7 (0.3, 1.6)	70.9 (63.5, 79.3)	0.3 (0.1, 0.8)	89.2 (81.0, 96.3)
	5.0/1000 PYAR	2.6 (1.1, 5.3)	59.3 (52.2, 66.5)	1.9 (0.7, 4.0)	70.5 (62.8, 79.0)	0.7 (0.1, 2.0)	89.0 (80.6, 96.2)
	10.0/1000 PYAR	5.1 (2.1, 10.4)	58.4 (51.0, 65.9)	3.7 (1.4, 7.9)	69.8 (61.6, 78.6)	1.4 (0.3, 4.0)	88.7 (79.9, 96.1)
ϕ = 0.5	0.5/1000 PYAR	0.3 (0.1, 0.6)	59.0 (52.1, 66.1)	0.2 (0.1, 0.4)	69.8 (62.2, 78.4)	0.1 (0.0, 0.2)	88.9 (80.4, 96.2)
	1.0/1000 PYAR	0.5 (0.2, 1.1)	58.9 (52.0, 66.1)	0.4 (0.1, 0.9)	69.7 (62.1, 78.4)	0.1 (0.0, 0.4)	88.8 (80.3, 96.2)
	2.0/1000 PYAR	1.1 (0.4, 2.2)	58.8 (51.8, 66.0)	0.8 (0.3, 1.7)	69.6 (61.8, 78.3)	0.3 (0.1, 0.9)	88.8 (80.1, 96.1)
	5.0/1000 PYAR	2.7 (1.1, 5.4)	58.3 (51.1, 65.6)	2.0 (0.7, 4.3)	69.1 (61.1, 78.1)	0.7 (0.1, 2.1)	88.6 (79.7, 96.1)
	10.0/1000 PYAR	5.2 (2.2, 10.5)	57.5 (49.9, 65.1)	3.9 (1.5, 8.4)	68.4 (59.9, 77.6)	1.4 (0.3, 4.2)	88.2 (78.8, 96.0)

PYAR: person-years at risk (incidence rate denominator).

[a]Case probabilities refer to the likelihood that at least one symptomatic cholera case occurs in the community. Effectiveness is defined as the reduction in this probability relative to its estimate under status quo protocols.

[b]Estimates are reported as median (95% CrI), as obtained via bootstrap resampling.
