# Supplementary material for: Strategies to Prevent Cholera Introduction during International Personnel Deployments: A Computational Modeling Analysis Based on the 2010 Haiti Outbreak
Source: PLoS Med. 2016 Jan 26;13(1):e1001947. doi: 10.1371/journal.pmed.1001947 (PMC4727895; doi:10.1371/journal.pmed.1001947)
Supplement: S13 Table — (PDF) [file pmed.1001947.s013.pdf]

S13 Table. Sensitivity analysis: basic reproductive numbers (R_0) with varying relative infectiousness of symptomatic cases.

Modeled infectiousness	Initial conditions		Basic reproduction number (R_0) estimate[a,b]			
			Geographical area		Transmission pathway	
			Artibonite communes	Nationwide	River-mediated	Local
$r = 1 + 0.5\text{Log}_{10}(v)$	Number of infected arrivals	1	1.81 (1.66, 2.03)	1.15 (1.00, 1.37)	30.29 (30.08, 30.49)	1.08 (0.93, 1.30)
		2	1.60 (1.47, 1.78)	0.98 (0.86, 1.16)	26.65 (26.47, 26.82)	0.92 (0.80, 1.10)
		3	1.51 (1.39, 1.68)	0.92 (0.80, 1.09)	24.67 (24.51, 24.83)	0.86 (0.73, 1.03)
	Background cholera incidence rate	0.5/1000 PYAR	1.81 (1.66, 2.02)	1.15 (1.00, 1.37)	30.27 (30.06, 30.47)	1.08 (0.93, 1.30)
		1.0/1000 PYAR	1.81 (1.66, 2.02)	1.15 (1.00, 1.36)	30.25 (30.05, 30.45)	1.08 (0.93, 1.30)
		2.0/1000 PYAR	1.81 (1.66, 2.02)	1.15 (1.00, 1.36)	30.22 (30.01, 30.43)	1.08 (0.93, 1.30)
		5.0/1000 PYAR	1.80 (1.66, 2.01)	1.14, 1.00, 1.35)	30.13 (29.91, 30.35)	1.08 (0.93, 1.28)
		10.0/1000 PYAR	1.80 (1.66, 1.99)	1.14 (0.99, 1.33)	29.99 (29.67, 30.24)	1.07 (0.93, 1.27)
$r = 1 + 2\text{Log}_{10}(v)$	Number of infected arrivals	1	1.77 (1.61, 1.98)	1.11 (0.95, 1.31)	30.30 (30.11, 30.51)	1.04 (0.88, 1.25)
		2	1.56 (1.41, 1.74)	0.94 (0.79, 1.13)	26.67 (26.50, 26.84)	0.88 (0.73, 1.07)
		3	1.48 (1.35, 1.66)	0.89 (0.76, 1.06)	24.68 (24.52, 24.84)	0.83 (0.70, 1.01)
	Background cholera incidence rate	0.5/1000 PYAR	1.77 (1.61, 1.98)	1.11 (0.95, 1.31)	30.29 (30.09, 30.50)	1.04 (0.88, 1.25)
		1.0/1000 PYAR	1.77 (1.61, 1.98)	1.11 (0.95, 1.31)	30.27 (30.08, 30.48)	1.04 (0.88, 1.25)
		2.0/1000 PYAR	1.77 (1.61, 1.97)	1.10 (0.95, 1.31)	30.24 (30.04, 30.45)	1.04 (0.88, 1.24)
		5.0/1000 PYAR	1.76 (1.60, 1.96)	1.10 (0.95, 1.30)	30.15 (29.93, 30.37)	1.04 (0.88, 1.23)
		10.0/1000 PYAR	1.75 (1.60, 1.94)	1.09 (0.95, 1.28)	30.01 (29.70, 30.27)	1.03 (0.88, 1.22)

PYAR: person-years at risk (incidence rate denominator).

[a]R_0 estimates are reported as median (95% CrI) from the posterior distribution of the parameters fitted under assumptions of one, two, or three infected peacekeepers at the outset of the epidemic. We derive the formula for R_0 via the next-generation matrix approach (S1 Text §3.9).

[b]Incidence-rate-specific R_0 estimates are obtained by pooling estimates across parameter sets according to the binomial probabilities of one, two, or three infected peacekeepers arriving (S1 Text §1.1).
