# Supplementary material for: Strategies to Prevent Cholera Introduction during International Personnel Deployments: A Computational Modeling Analysis Based on the 2010 Haiti Outbreak
Source: PLoS Med. 2016 Jan 26;13(1):e1001947. doi: 10.1371/journal.pmed.1001947 (PMC4727895; doi:10.1371/journal.pmed.1001947)
Supplement: S14 Table — (PDF) [file pmed.1001947.s014.pdf]

S14 Table. Sensitivity analysis: case probabilities with varying relative infectiousness of symptomatic cases in local transmission.

Modeled infectiousness	Infectious arrivals	Status quo	Antimicrobial chemoprophylaxis (ACP)		Oral cholera vaccine (OCV)		Combined ACP and OCV	
		Case probability (%)[a,c]	Case probability (%)[a,c]	Effectiveness (%)[b,c]	Case probability (%)[a,c]	Effectiveness (%)[b,c]	Case probability (%)[a,c]	Effectiveness (%)[b,c]
$r = 1 + 0.5\text{Log}_{10}(v)$	1	81.5 (80.5, 82.6)	68.0 (66.7, 69.3)	16.6 (14.7, 18.5)	31.4 (30.2, 32.7)	61.5 (59.8, 63.1)	18.1 (17.1, 19.2)	77.7 (76.4, 79.1)
	2	97.1 (96.6, 97.6)	89.3 (88.4, 90.1)	8.0 (7.0, 9.0)	47.8 (46.4, 49.1)	50.8 (49.4, 52.3)	29.5 (28.3, 30.8)	69.6 (68.3, 70.9)
	3	99.4 (99.2, 99.6)	96.0 (95.5, 96.5)	3.4 (2.8, 4.0)	60.7 (59.4, 62.1)	38.9 (37.6, 40.3)	38.7 (37.4, 40.1)	61.1 (59.7, 62.4)
$r = 1 + 2\text{Log}_{10}(v)$	1	82.7 (81.6, 83.7)	68.6 (67.3, 70.0)	17.0 (15.1, 18.9)	31.1 (29.9, 32.4)	62.3 (60.7, 63.9)	16.9 (15.9, 18.0)	79.5 (78.2, 80.8)
	2	96.7 (96.2, 97.2)	89.3 (88.4, 90.2)	7.6 (6.6, 8.6)	48.5 (47.1, 49.9)	49.8 (48.3, 51.3)	29.1 (27.8, 30.3)	69.9 (68.6, 71.3)
	3	99.4 (99.2, 99.6)	96.2 (95.7, 96.8)	3.2 (2.6, 3.8)	61.4 (60.0, 62.8)	38.2 (36.8, 39.6)	37.4 (36.1, 38.8)	62.4 (61.0, 63.7)

[a]The probability outcome measure is defined as the likelihood for at least one case to occur in the community following arrival of 1, 2, or 3 infected peacekeepers.
[b]Effectiveness is defined as the reduction in this probability relative to its estimate under status quo.
[c]Estimates are reported as median (95% CrI), as obtained via bootstrap resampling.
