# Supplementary material for: Strategies to Prevent Cholera Introduction during International Personnel Deployments: A Computational Modeling Analysis Based on the 2010 Haiti Outbreak
Source: PLoS Med. 2016 Jan 26;13(1):e1001947. doi: 10.1371/journal.pmed.1001947 (PMC4727895; doi:10.1371/journal.pmed.1001947)
Supplement: S15 Table — (PDF) [file pmed.1001947.s015.pdf]

S15 Table. Sensitivity analysis: fitted parameters with varying relative infectiousness of symptomatic cases.

Parameter	Definition	Infectious arrivals	$r = 1 + \mathrm{Log}_{10}(v)$	$r = 1 + 0.5\mathrm{Log}_{10}(v)$	$r = 1 + 2\mathrm{Log}_{10}(v)$
π	Symptom probability (*local transmission*)[a]	1	0.241 (0.219, 0.267)	0.243 (0.220, 0.267)	0.241 (0.218, 0.270)
		2	0.239 (0.214, 0.267)	0.241 (0.218, 0.269)	0.242 (0.216, 0.266)
		3	0.238 (0.213, 0.264)	0.239 (0.215, 0.269)	0.242 (0.215, 0.266)
$\mathrm{Log}_{10}(\beta_W)$	Environmental *V. cholerae* exposure rate[a]	1	–6.665 (–6.676, –6.653)	–6.664 (–6.678, –6.654)	–6.664 (–6.676, –6.652)
		2	–6.743 (–6.755, –6.733)	–6.743 (–6.754, –6.733)	–6.744 (–6.752, –6.734)
		3	–6.792 (–6.804, –6.781)	–6.792 (–6.804, –6.781)	–6.792 (–6.803, –6.781)
β_L	Contact rate with *V. cholerae* carriers[a]	1	0.219 (0.184, 0.260)	0.230 (0.199, 0.276)	0.204 (0.172, 0.244)
		2	0.184 (0.156, 0.222)	0.196 (0.171, 0.233)	0.172 (0.143, 0.208)
		3	0.170 (0.146, 0.205)	0.182 (0.157, 0.218)	0.162 (0.137, 0.197)
k	Asymptotic transmission constant[a]	1	1201 (1010, 1413)	1275 (1082, 1496)	1045 (897, 1240)
		2	1456 (1149, 1845)	1559 (1231, 1867)	1219 (1013, 1573)
		3	1644 (1242, 1954)	1775 (1300, 2123)	1252 (1072, 1620)
$\mathrm{Log}_{10}(\omega)$	Migration rate (d^{-1})[a]	1	–1.314 (–1.331, –1.29)	–1.313 (–1.332, –1.297)	–1.313 (–1.330, –1.295)
		2	–1.332 (–1.349, –1.316)	–1.333 (–1.349, –1.317)	–1.332 (–1.347, –1.317)
		3	–1.344 (–1.362, –1.327)	–1.343 (–1.362, –1.327)	–1.344 (–1.361, –1.326)
$\mathrm{Log}_{10}(N^{(W)}/N^{(A)})$	Proportion exposed to Artibonite (*of Artibonite-adjacent communes*)[a]	1	–1.618 (–1.630, –1.608)	–1.619 (–1.629, –1.607)	–1.619 (–1.630, –1.608)
		2	–1.596 (–1.606, –1.585)	–1.596 (–1.605, –1.585)	–1.595 (–1.605, –1.587)
		3	–1.580 (–1.591, –1.569)	–1.581 (–1.591, –1.569)	–1.580 (–1.591, –1.570)

[a]Estimates are reported as median (95% CrI) describing the distribution sampled by Markov Chain Monte Carlo.
