# Supplementary material for: Strategies to Prevent Cholera Introduction during International Personnel Deployments: A Computational Modeling Analysis Based on the 2010 Haiti Outbreak
Source: PLoS Med. 2016 Jan 26;13(1):e1001947. doi: 10.1371/journal.pmed.1001947 (PMC4727895; doi:10.1371/journal.pmed.1001947)
Supplement: S16 Table — (PDF) [file pmed.1001947.s016.pdf]

S16 Table. Sensitivity analysis: case probabilities with varying vaccine protection against infection.

Outcome measure (%)[a,b]	Background cholera incidence rate	Status quo	Vaccine efficacy against infection[d]			
			5%	10%	25%	50%
Case probability	0.5/1000 PYAR	0.7 (0.3, 1.3)	0.2 (0.1, 0.5)	0.2 (0.1, 0.4)	0.2 (0.1, 0.4)	0.1 (0.06, 0.2)
	1.0/1000 PYAR	1.3 (0.6, 2.5)	0.5 (0.2, 0.9)	0.5 (0.2, 0.9)	0.4 (0.2, 0.7)	0.3 (0.1, 0.5)
	**1.8/1000 PYAR[c]**	**2.3 (1.1, 4.5)**	**0.9 (0.4, 1.7)**	**0.8 (0.4, 1.6)**	**0.7 (0.3, 1.3)**	**0.5 (0.2, 0.9)**
	2.0/1000 PYAR	2.6 (1.2, 5.0)	1.0 (0.4, 1.9)	0.9 (0.4, 1.8)	0.8 (0.4, 1.5)	0.5 (0.2, 1.0)
	5.0/1000 PYAR	6.4 (3.0, 11.9)	2.4 (1.1, 4.6)	2.3 (1.0, 4.3)	1.9 (0.9, 3.6)	1.3 (0.6, 2.4)
	10.0/1000 PYAR	12.4 (5.8, 22.4)	4.7 (2.2, 8.8)	4.5 (2.1, 8.4)	3.8 (1.7, 7.1)	2.5 (1.2, 4.8)
Effectiveness	0.5/1000 PYAR		62.9 (61.3, 64.5)	64.8 (63.3, 66.3)	70.7 (69.4, 71.9)	80.4 (79.6, 81.3)
	1.0/1000 PYAR		62.8 (61.2, 64.4)	64.8 (63.3, 66.3)	70.6 (69.4, 71.9)	80.4 (79.6, 81.2)
	**1.8/1000 PYAR[c]**		**62.7 (61.1, 64.3)**	**64.7 (63.2, 66.2)**	**70.5 (69.3, 71.8)**	**80.3 (79.5, 81.2)**
	2.0/1000 PYAR		62.7 (61.1, 64.3)	64.6 (63.1, 66.1)	70.5 (69.2, 71.8)	80.3 (79.4, 81.1)
	5.0/1000 PYAR		62.3 (60.7, 63.9)	64.3 (62.7, 65.8)	70.1 (68.8, 71.4)	80.0 (79.1, 80.9)
	10.0/1000 PYAR		61.7 (60.0, 65.3)	63.6 (61.9, 65.3)	69.5 (68.0, 71.0)	79.5 (78.3, 80.5)

PYAR: person-years at risk (incidence rate denominator).

[a]Case probabilities refer to the likelihood that at least one symptomatic cholera case occurs in the community. Effectiveness is defined as the reduction in this probability relative to its estimate under status quo protocols.

[b]Estimates are reported as median (95% CrI), as obtained via bootstrap resampling.

[c]The incidence of cholera among adults in Nepal was previously estimated to be 1.8/1000 PYAR [1].

[d]Protection against infection is modeled as a proportional reduction in the probability for a peacekeeper to be infected upon departing Haiti. The analysis considers baseline estimates of vaccine reductions in bacterial shedding as indicated in Table 3 (Φ = 0.0194) [11].
