# Supplementary material for: Strategies to Prevent Cholera Introduction during International Personnel Deployments: A Computational Modeling Analysis Based on the 2010 Haiti Outbreak
Source: PLoS Med. 2016 Jan 26;13(1):e1001947. doi: 10.1371/journal.pmed.1001947 (PMC4727895; doi:10.1371/journal.pmed.1001947)
Supplement: S1 Text — (PDF) [file pmed.1001947.s017.pdf]

Supplemental methods

Strategies to prevent cholera introduction during international personnel deployments: a computational modeling analysis based on the 2010 Haiti outbreak

Joseph A. Lewnard[1,*], Marina Antillón[1,2], Gregg Gonsalves[1,2,3], Alice M. Miller[2,3,4], Albert I. Ko[1,2,5], and Virginia E. Pitzer[1]

[1] *Department of Epidemiology of Microbial Diseases, Yale School of Public Health, New Haven, Connecticut, United States*
[2] *Global Health Justice Partnership, Yale University, New Haven, Connecticut, United States*
[3] *Yale Law School, New Haven, Connecticut, United States*
[4] *Department of Social and Behavioral Medicine, Yale School of Public Health, New Haven, Connecticut, United States*
[5] *Centro de Pesquisas Gonçalo Moniz, Fundação Oswaldo Cruz, Ministério da Saúde, Salvador, Brazil*
[*] ***Address correspondence to:*** *joseph.lewnard@yale.edu*

Contents

S1 Cholera importation probability **3**
S1.1 Overview 3
S1.2 Symptom probability in endemic settings 4
S1.3 Asymptomatic carriage from endemic transmission 5
S1.4 Cholera progression during transit 5

S2 Intervention measures **7**
S2.1 Overview 7
S2.2 Rapid diagnostic screening 8
S2.3 Antimicrobial chemoprophylaxis 9
S2.3.1 Time-of-departure prophylaxis 10
S2.3.2 Early-initiated prophylaxis 11
S2.4 Oral cholera vaccine 12
S2.5 Benefit to peacekeepers: reduction in disease risk 12
S2.5.1 Antimicrobial chemoprophylaxis 13
S2.5.2 Oral cholera vaccination 13
S2.5.3 Combined chemoprophylaxis and immunization 13

S3 Model description **14**
S3.1 Disease transitions 14
S3.2 Force of infection 15
S3.3 Heterogeneous infectivity 16
S3.4 Clinical outcomes of infection 17
S3.5 Peacekeeping compartments 17
S3.6 Population structure and migration 17
S3.7 Model equations 19
S3.8 Stochastic implementation 21
S3.9 Reproductive numbers 21

S4 Model calibration **23**
S4.1 Parameter estimation and multimodel inference 23
S4.2 Prior distributions 23
S4.3 Markov chain Monte Carlo sampling 25
S4.4 Timing of the first case 26

S5 References **27**

Introduction

The severe cholera epidemic introduced to Haiti in 2010 underscores the risk for infectious diseases to spread internationally in the context of large-scale personnel movements. We assessed how implementing several biomedical interventions prior to peacekeepers' deployment from Nepal could have impacted the likelihood for epidemic cholera in Haiti. These included screening for *Vibrio cholerae* carriage using rapid diagnostic tests (RDTs); administering antimicrobial chemotherapies following prophylactic schedules beginning at or one week before departure; and immunizing peacekeepers with oral cholera vaccines (OCVs) beginning five weeks before departure, with or without antimicrobial chemoprophylaxis in conjunction. We defined as status quo the current policy, in place as of 2010, of implementing no interventions among persons who do not show cholera symptoms.

We approached the question by addressing how the interventions could have impacted the probability for either or both of two events requisite to the establishment of an epidemic:

1. Undetected importation of *Vibrio cholerae* from the endemic source country (Nepal) to Haiti by an asymptomatically-infected peacekeeper; and
2. Transmission of *V. cholerae* from asymptomatic peacekeepers to the general population.

We provide a detailed description of our approach to quantifying the probabilities for these events in this Supplement. We outline the circumstances leading to cholera importation in Section S1. We describe the interventions, the rationale for their consideration, and the associated mechanisms of protection in Section S2. We lay out the model for cholera transmission in Haiti in Section S3, followed by a description of the approach taken to calibrate the model to dynamics of the Haiti epidemic in Section S4. Unless indicated otherwise, numerical results in this supplement are presented in the form median [95% credible interval].

S1 Cholera importation probability

S1.1 Overview

Since cholera had been absent from Haiti prior to the 2010 outbreak, the occurrence of cases in our model depended on the arrival of infected peacekeepers. Prior to departing for Haiti, peacekeepers had been given a ten-day free period to visit their families, during which they were likely exposed to cholera transmission within the general population of Nepal [1]. The number of peacekeepers who were infected at time of departure and arrival is unknown, as is the geographic source of infection within Nepal.

A peacekeeper's probability for transporting *V. cholerae* is inherently linked to the probability of being exposed to cholera in the source country, and thus to the background cholera incidence rate in Nepal. While this rate has been estimated at 1.8 cases per 1000 person-years at risk (PYAR) among adults [2], this estimate is based on limited data and does not take into account potential geographic and seasonal variation in risk. Such uncertainty has implications for evaluating the effectiveness of interventions because the relation between peacekeepers' individual risk for infection and the probability for any peacekeeper within a battalion to carry *V. cholerae* is non-linear. Consequently, we evaluated intervention effectiveness across incidence rates ranging from 0.5 to 10.0 cases per 1000 PYAR.

We accounted for cholera progression during transit from Nepal to the MINUSTAH base and modeled the number of peacekeepers arriving in the infected state under status quo as a Binomially-distributed random variable

$$X_0 \sim \text{Binom}(n, p),$$

where $n = 454$ specified the number of peacekeepers deployed to the MINUSTAH base in October 2010 [1], and p specified the estimated prevalence of infection among those who arrived (S1.4). The Binomial distribution assumes each peacekeeper's infection status is independent. Here this assumption was motivated by the fact that while visiting their families across Nepal, peacekeepers would have acquired infection independently of one another's outcomes.

Under status quo, we defined $p = p_0$, accounting for prevalence among arrivals absent any intervention to limit carriage. We modeled rapid diagnostic screening and time-of-departure and early-initiated antimicrobial chemoprophylaxis interventions resulting in prevalences p_{RDT}, $p_{\text{Abx}}^{(\text{a})}$, and $p_{\text{Abx}}^{(\text{b})}$, respectively.

S1.2 Symptom probability in endemic settings

We computed the probability σ for symptomatic carriage in South Asian endemic countries using data from epidemiological surveys monitoring onset of symptomatic and asymptomatic infection among contacts of cholera patients. Inclusion criteria for the meta-analysis were that studies:

1. Were undertaken in South Asian endemic countries;
2. Assessed whether subjects were *V. cholerae*–culture-negative at baseline; and
3. Presented data from contacts infected with the El Tor biotype of *V. cholerae*.

We identified studies by searching the phrase "(household OR family OR community) AND contact AND index AND cholera AND symptom*" in PubMed. The procedure for pooling effect sizes is described in the Main Text.

S1.3 Asymptomatic carriage from endemic transmission

We estimated the probability for a peacekeeper to acquire asymptomatic cholera infection via exposure to endemic transmission conditions typical to Nepal. Taking our meta-analytic estimate of symptom probability in an endemic setting (σ = 24.2% [95% CI: 14.4%, 40.7%]; Main Text), and letting the background incidence rate take the values

$$\iota = \left(\tfrac{0.5}{1000} \quad \tfrac{1.0}{1000} \quad \tfrac{\mathbf{1.8}}{\mathbf{1000}} \quad \tfrac{2.0}{1000} \quad \tfrac{5.0}{1000} \quad \tfrac{10.0}{1000} \right),$$

asymptomatic infections occurred at the rate $\iota(1-\sigma)\sigma^{-1}$. Defining $1/\delta = 1.55$ days as the incubation period (Table 1), the prevalence of incubation-phase asymptomatic infection among departures was

$$\begin{aligned}\frac{E_A(\tau)}{N_K} &= \iota \left(\frac{1-\sigma}{\sigma}\right)\left(\frac{1/\delta}{365.25}\right)\\ &= 0.7 \text{ [0.3, 1.3] per 100,000 (lower bound)}\\ &= 2.4 \text{ [1.1, 4.6] per 100,000 (WHO est.)}\\ &= 13.4 \text{ [6.1, 25.8] per 100,000 (upper bound)}\end{aligned}$$

For a duration of shedding equal to $1/\gamma_A$ days (Table 1), the prevalence of asymptomatic shedding among departures was estimated as

$$\begin{aligned}\frac{I_A(\tau)}{N_K} &= \iota \left(\frac{1-\sigma}{\sigma}\right)\left(\frac{1/\gamma_A}{365.25}\right)\\ &= 2.2 \text{ [1.0, 4.2] per 100,000 (lower bound)}\\ &= 7.9 \text{ [3.6, 15.3] per 100,000 (WHO est.)}\\ &= 44.0 \text{ [20.1, 84.8] per 100,000 (upper bound)}\end{aligned}$$

Full results across incidence rates are presented in Table S2.

S1.4 Cholera progression during transit

To account for marked individual-level variability in the durations of cholera incubation, carriage, and symptoms [11,12, Table 2 (Main Text)], we modeled the time individuals spent in the various infected classes as exponentially-distributed random variables, letting $f_\lambda(t)$ and $F_\lambda(t)$ denote the probability density and cumulative distribution functions, respectively, evaluated at t for the exponential distribution with rate λ. We allowed for a two-day transit time between Nepal and the MINUSTAH base [1,13], and defined the probability that a peacekeeper would depart Nepal and arrive in Haiti experiencing any particular states at time τ and $\tau + 2$, respectively, as the product

$$\mathbb{P}(\text{Departure state}) \times \mathbb{P}(\text{Arrival state}|\text{Departure state})$$

In each case, the probability of the departure state was the prevalence of that state within the population as estimated above (Section S1.3). The conditional

probability of the arrival state was inferred from the exponentially-distributed durations of incubation and carriage, as detailed below:

1. Peacekeepers departed and arrived in the E_A state with probability

$$\begin{aligned}
&\mathbb{P}(E_A) \times \mathbb{P}(\text{No progression to } I_A) \\
&= \frac{E_A(\tau)}{N_K}\Big(1 - F_\delta(2)\Big) \\
&= 0.2 \ [0.1,\ 0.4] \text{ per } 100{,}000 \text{ (lower bound)} \\
&= 0.7 \ [0.3,\ 1.3] \text{ per } 100{,}000 \text{ (WHO est)} \\
&= 3.7 \ [1.7,\ 7.1] \text{ per } 100{,}000 \text{ (upper bound)}
\end{aligned}$$

2. Peacekeepers departed in the E_A state and arrived in the I_A state with probability

$$\begin{aligned}
&\mathbb{P}(E_A) \times \int_0^2 \mathbb{P}(\text{Progression to } I_A \text{ at time } t)\mathbb{P}(\text{No progression to } R|t)\mathrm{d}t \\
&= \frac{E_A(\tau)}{N_K}\int_0^2 f_\delta(x)\Big(1 - F_{\gamma_A}(2-t)\Big)\mathrm{d}t \\
&= \frac{E_A(\tau)}{N_K}\frac{\delta(1-e^{-2\gamma_A}) - \gamma_A(1-e^{-2\delta})}{\delta - \gamma_A} \\
&= 0.1 \ [0.0,\ 0.2] \text{ per } 100{,}000 \text{ (lower bound)} \\
&= 0.4 \ [0.2,\ 0.7] \text{ per } 100{,}000 \text{ (WHO est.)} \\
&= 2.0 \ [0.9,\ 3.9] \text{ per } 100{,}000 \text{ (upper bound)}
\end{aligned}$$

3. Peacekeepers departed and arrived in the I_A state with probability

$$\begin{aligned}
&\mathbb{P}(I_A) \times \mathbb{P}(\text{No progression to } R) \\
&= \frac{I_A(\tau)}{N_K}\Big(1 - F_{\gamma_A}(2)\Big) \\
&= 1.5 \ [0.7,\ 2.9] \text{ per } 100{,}000 \text{ (lower bound)} \\
&= 5.3 \ [2.4,\ 10.3] \text{ per } 100{,}000 \text{ (WHO est.)} \\
&= 29.7 \ [13.6,\ 57.3] \text{ per } 100{,}000 \text{ (upper bound)}
\end{aligned}$$

In total,

$$\begin{aligned}
p_0 &= \frac{E_A(\tau)}{N_K}\left(e^{-2\delta} + \frac{\delta(1-e^{-2\gamma_A}) - \gamma_A(1-e^{-2\delta})}{\delta - \gamma_A}\right) + \frac{I_A(\tau)}{N_K}e^{-2\gamma_A} \\
&= 1.8 \ [0.8,\ 3.4] \text{ per } 100{,}000 \text{ (lower bound)} \\
&= 6.4 \ [2.9,\ 12.3] \text{ per } 100{,}000 \text{ (WHO est.)} \\
&= 35.4 \ [16.2,\ 68.2] \text{ per } 100{,}000 \text{ (upper bound)}
\end{aligned}$$

The majority of persons incubating cholera at time τ were expected to shed after two days in transit ($F_\delta(2) = 72\%$), while over half of those remaining in the incubation stage upon arrival were expected to begin shedding within the first day at the MINUSTAH base. We consequently assumed all infected peacekeepers shed *V. cholerae* upon arrival when the number of infected arrivals exceeded one (probability $< 10^{-4}$).

S2 Intervention measures

S2.1 Overview

We considered four interventions that could be undertaken among peacekeepers in advance of arrival to limit disease introduction risk: (1) screening with RDTs to ascertain potential *V. cholerae* carriers; (2) administering prophylactic antibiotics to prevent shedding onset and limit shedding duration; (3) immunizing with OCV; and (4) immunizing with OCV combined with chemoprophylaxis. We list parameters used to model the interventions in Table 3 (Main Text).

We assessed RDTs as the likely screening method because gold-standard culture-based approaches require laboratory facilities that are unavailable in many cholera-endemic settings. Furthermore, culturing results in a 24 to 72 h delay between rectal swabbing and diagnosis, during which individuals could acquire and transmit infection among one another, exacerbating risk for cholera importation [14–16]. Although typically used for diarrheal stool samples, rapid tests are capable of detecting *V. cholerae* on rectal swabs if samples are incubated for 4–6h in alkaline peptone water [17] even when the total vibrio count prior to enrichment is as low as 200 colony-forming units (cfu) [18]. We inferred test performance from previous estimates of RDT sensitivity and specificity, as applied to enriched rectal-swab samples [17]. Sensitivity estimates provided the probability for testing to identify peacekeepers shedding *V. cholerae* at their time of deployment, while specificity estimates gave the proportion of tests expected to produce false positive outcomes. We assumed tests would not detect latent, incubation-phase infections preceding shedding onset.

Numerous antimicrobial drugs are efficacious for the purposes of cholera treatment and prophylaxis. Optimal drugs for use in any setting depend upon the local *V. cholerae* resistance profile [19]. Consequently, we used estimates from meta-analyses aggregated across antimicrobial drug classes to quantify two correlates of prophylactic efficacy: (1) the probability that prophylaxed, cholera-exposed individuals would clear infection prior to shedding onset; and (2) reductions in the duration of shedding among prophylaxed individuals. We considered two drug administration schedules. The first was a typical prophylaxis schedule begun at the time of departure for Haiti ("time-of-departure prophylaxis"), intended to hasten clearance of infection among peacekeepers who had acquired infection during the leave period. Shown to be particularly

effective in an early trial [20], the second ("early-initiated prophylaxis") was begun seven days prior to deployment and was designed to hasten clearance as well as prevent *V. cholerae* acquisition leading up to departure.

Although OCVs do not necessarily protect recipients against infection [21,22], they reduce shedded *V. cholerae* colony forming units per milliliter (cfu mL^{-1}) among vaccinees who experience infection [21]. We accounted for this correlate of protection in comparing transmission risk for immunized and non-immunized peacekeepers who experience asymptomatic infection, assuming peacekeepers received two doses of killed whole-cell vaccine without recombinant B subunit (Shanchol^{TM}) at five and three weeks prior to deployment [28, Figure 1 in Main Text].

S2.2 Rapid diagnostic screening

Defining the test sensitivity (Se) as the conditional probability for a positive test result given that an individual was shedding *V. cholerae*, we inferred the distribution to be $\text{Se} \sim \text{Beta}(65, 3)$ (median 96.1% [95% CI 89.4%, 99.1%]) based on the 65 RDT-positive, culture-positive and three RDT-negative, culture-positive outcomes in [17]. Defining the test specificity (Sp) as the conditional probability for a negative test result given that an individual did not shed *V. cholerae*, we inferred the distribution $\text{Sp} \sim \text{Beta}(61, 5)$ (median 92.8% [85.1%, 97.4%]) based on the 61 RDT-negative, culture-negative and five RDT-positive, culture-negative results in [17].

We defined the number of peacekeepers who arrive and proceed to shed asymptomatically as

$$X_{\text{RDT}} \sim \text{Binom}(n = 454, p = p_{\text{RDT}}),$$

where we derived p_{RDT} considering that infected peacekeepers left for Haiti under either of two scenarios:

1. Departure in the E_A state, where the test correctly identified the peacekeeper was not shedding with probability Sp;
2. Departure in the I_A state, where the test failed to detect shedding with probability $(1 - \text{Se})$.

As detailed in Section S1.4, we computed the probability for arriving infected by integrating over two days to account for $E_A \to I_A$ and $I_A \to R$ transitions, here incorporating test sensitivity and specificity into the state-specific departure

probabilities:

$$
\begin{aligned}
p_{\mathrm{RDT}} &= \mathrm{Sp}\frac{E_A(\tau)}{N_K}\left(e^{-2\delta}+\frac{\delta(1-e^{-2\gamma_A})-\gamma_A(1-e^{-2\delta})}{\delta-\gamma_A}\right)+(1-\mathrm{Se})\frac{I_A(\tau)}{N_K}e^{-2\gamma_A}\\
&= 0.3\ [0.1,\ 0.7] \text{ per 100,000 (lower bound)}\\
&= 1.2\ [0.5,\ 2.4] \text{ per 100,000 (WHO est.)}\\
&= 6.5\ [2.9,\ 13.1] \text{ per 100,000 (upper bound)}
\end{aligned}
$$

We defined a Binomially-distributed random variable Z as the number of false-positive test outcomes. Such outcomes arose under either of two circumstances:

1. Shedding was detected erroneously with probability $(1-\mathrm{Sp})$ in uninfected peacekeepers, who comprised a proportion

$$1-\frac{E_A(\tau)+I_A(\tau)}{N_K}$$

   of the total number of peacekeepers deployed; or

2. Shedding was detected erroneously with probability $(1-\mathrm{Sp})$ in an infected peacekeeper experiencing incubation (E_A) prior to shedding onset.

False positive outcomes of the second type were protective against cholera importation. Consequently, we considered only the first class of false positive outcomes in defining, as an adverse consequence of screening,

$$
\begin{aligned}
Z &\sim \mathrm{Binom}\left(n=454, p=(1-\mathrm{Sp})\left(1-\frac{E_A(\tau)+I_A(\tau)}{N_K}\right)\right)\\
&= 32\ [10,\ 70] \text{ (for all background incidence estimates; Table S5).}
\end{aligned}
$$

S2.3 Antimicrobial chemoprophylaxis

Numerous studies have demonstrated that antimicrobial drugs are efficacious at preventing shedding onset in cholera-exposed persons. A previous meta-analytic estimate [32] provided the proportion $(1-\upsilon_{\mathrm{Abx}}) = 66\%$ [34–82%] of infections averted by chemoprophylaxis among cholera-exposed individuals not shedding at baseline. Here υ_{Abx} was the ratio of the proportions of contacts of cholera cases who experienced *V. cholerae* shedding in treatment and control groups,

$$\frac{\mathbb{P}_{\mathrm{Treatment}}(\mathrm{Shed}|\mathrm{Exposed})}{\mathbb{P}_{\mathrm{Control}}(\mathrm{Shed}|\mathrm{Exposed})}.$$

We modeled that this mechanism of protection prevented shedding onset in a proportion υ_{Abx} of individuals who would otherwise experience shedding.

A second mechanism of protection afforded by antimicrobial therapies is a reduction in the duration of *V. cholerae* shedding [33]. This effect has been documented in prophylaxis trials [20,34], including among cholera-exposed persons

who were culture-negative at baseline and experienced shedding onset following drug receipt [34]. Because data from prophylaxis trials were sparse for inferring the reduction in duration of pathogen excretion, we used meta-analytic estimates [33] of the reduction in the duration of pathogen excretion among symptomatic cases receiving antimicrobial drugs. This estimated effect size (2.74 d [2.40–3.07]) was conservative relative to findings from the prophylaxis trials [Table 3 in 34].

To propagate uncertainty about intervention efficacy in model realizations, we fitted distributions for the effect sizes by minimizing summed squared errors relative to reported means and 95% confidence interval quantiles:

$$\upsilon_{\text{Abx}} \sim \text{LogNorm}(-1.07,\ 0.33)$$

$$(\gamma_A^{-1} - \gamma_{\text{Abx}}^{-1}) \sim \text{Norm}(2.74,\ 0.17).$$

To investigate how real-world limitations such as reduced antimicrobial susceptibility or noncompliance with treatment could influence the effectiveness of time-of-departure and early-initiated chemoprophylaxis interventions, we conducted additional analyses assessing outcomes in the context of 10% to 50% reduced protection against shedding onset and 10% to 50% longer time to bacterial clearance (Tables S6, S8).

S2.3.1 Time-of-departure prophylaxis

We compared two intervention timelines for antimicrobial prophylaxis. For time-of-departure prophylaxis ("a"), we considered administering drugs to peacekeepers beginning at deployment (two days before arrival at MINUSTAH). This prevented a proportion $(1-\upsilon_{\text{Abx}})$ of peacekeepers deployed in the E_A state from progressing to shedding. For those who experienced shedding due to deployment in the I_A state or progression $E_A \to I_A$ despite drug receipt, we modeled clearance at the rate γ_{Abx}. We defined the number of peacekeepers arriving infected as

$$X_{\text{Abx}}^{(\text{a})} \sim \text{Binom}(n = 454, p = p_{\text{Abx}}^{(\text{a})})$$

where we accounted for limited progression from $E_A \to I_A$ for those who arrived in the exposed state, and accelerated clearance of infection for those who arrived in or progressed to the I_A state. Following Section S1.4 above,

$$\begin{aligned} p_{\text{Abx}}^{(\text{a})} &= \upsilon_{\text{Abx}} \frac{E_A(\tau)}{N_K} \left(e^{-2\delta} + \frac{\delta(1 - e^{-2\gamma_{\text{Abx}}}) - \gamma_{\text{Abx}}(1 - e^{-2\delta})}{\delta - \gamma_{\text{Abx}}} \right) + \frac{I_A(\tau)}{N_K} e^{-2\gamma_{\text{Abx}}} \\ &= 1.1\ [0.4,\ 2.3] \text{ per } 100{,}000 \text{ (lower bound)} \\ &= 3.9\ [1.6,\ 8.2] \text{ per } 100{,}000 \text{ (WHO est.)} \\ &= 21.5\ [8.8,\ 45.5] \text{ per } 100{,}000 \text{ (upper bound)} \end{aligned}$$

The full results are presented in Table S2.

The absence of *V. cholerae* from MINUSTAH camp sewage and from carriage among UN personnel within two weeks of the start of the outbreak supports the notion that cholera transmission among peacekeepers was unlikely at the camp (see Discussion, Main Text). Moreover, the plane ride to Haiti would offer few modalities for interpersonal transmission. Consequently, we assumed transmission during transit and prophylactic protection against acquisition during transit contributed negligibly to the probability of arriving infected.

S2.3.2 Early-initiated prophylaxis

We modeled early-initiated prophylaxis ("b") beginning seven days prior to deployment and continuing during transit to Haiti. We accounted for reduced acquisition and accelerated clearance in estimating the prevalence of the asymptomatic infected states among peacekeepers following exposure to endemic transmission conditions. Modifying the formulae presented in Section S1.3,

$$\begin{aligned}
\frac{E_A^{(\mathrm{b})}(\tau)}{N_K} &= \upsilon_{\mathrm{Abx}}\iota\left(\frac{1-\sigma}{\sigma}\right)\left(\frac{1/\delta}{365.25}\right) \\
&= 0.2\ [0.1,\ 0.6] \text{ per 100,000 (lower bound)} \\
&= 0.8\ [0.3,\ 2.1] \text{ per 100,000 (WHO est.)} \\
&= 4.6\ [1.7,\ 11.6] \text{ per 100,000 (upper bound)} \\
\frac{I_A^{(\mathrm{b})}(\tau)}{N_K} &= \upsilon_{\mathrm{Abx}}\iota\left(\frac{1-\sigma}{\sigma}\right)\left(\frac{1/\gamma_{\mathrm{Abx}}}{365.25}\right) \\
&= 0.3\ [0.1,\ 1.0] \text{ per 100,000 (lower bound)} \\
&= 1.3\ [0.4,\ 3.5] \text{ per 100,000 (WHO est.)} \\
&= 7.0\ [2.3,\ 19.5] \text{ per 100,000 (upper bound)}
\end{aligned}$$

The full results are presented in Table S2.

We used the resulting prevalences of the $E_A^{(\mathrm{b})}$ and $I_A^{(\mathrm{b})}$ states to compute the probabilities for peacekeepers to have arrived infected, accounting for accelerated clearance during transit as in Section S2.3.1:

$$X_{\mathrm{Abx}}^{(\mathrm{b})} \sim \mathrm{Binom}\Big(n = 454, p = p_{\mathrm{Abx}}^{(\mathrm{b})}\Big)$$

for

$$\begin{aligned}
p_{\mathrm{Abx}}^{(\mathrm{b})} &= \frac{E_A^{(\mathrm{b})}(\tau)}{N_K}\left(e^{-2\delta} + \frac{\delta(1-e^{-2\gamma_{\mathrm{Abx}}}) - \gamma_{\mathrm{Abx}}(1-e^{-2\delta})}{\delta - \gamma_{\mathrm{Abx}}}\right) + \frac{I_A^{(\mathrm{b})}(\tau)}{N_K}e^{-2\gamma_{\mathrm{Abx}}} \\
&= 0.2\ [0.1,\ 0.6] \text{ per 100,000 (lower bound)} \\
&= 0.7\ [0.2,\ 2.3] \text{ per 100,000 (WHO est.)} \\
&= 3.9\ [1.1,\ 12.6] \text{ per 100,000 (upper bound)}
\end{aligned}$$

S2.4 Oral cholera vaccine

We used findings from a challenge trial to determine OCV-mediated mechanisms of protection [21]. In the trial, healthy, immunologically-naive North American volunteers were randomized to receive OCV (with or without the cholera toxin B-subunit) or placebo, and were subsequently challenged with a dose of 2×10^6 *V. cholerae* colony-forming units. All of 15 control subjects shedded *V. cholerae*, as did ten out of 11 recipients of OCV with B subunit and eight out of nine recipients of OCV without B subunit. This outcome suggested that although OCV reduced risk for disease, it did not limit risk for infection. This finding was subsequently replicated in a large field trial [22]. Existing protocols to isolate and treat diarrheal cases were assumed to limit transmission risk from peacekeepers experiencing symptomatic cholera under status quo. We consequently addressed reductions in *V. cholerae* concentration among asymptomatically infected individuals as the primary mechanism by which OCV would reduce transmission risk. We assessed the effect of any additional protection against infection (potentially applicable to next-generation vaccines) in a sensitivity analysis (Table S16).

In the challenge study, recipients of the killed whole vibrio vaccine without B subunit (Shanchol^{TM}) demonstrated a reduction in mean *V. cholerae* cfu mL^{-1}, shedding on average 9.9×10^5 cfu mL^{-1} versus 5.1×10^7 cfu mL^{-1} among controls. We defined this ratio (0.0194) of shedding in the vaccine versus control arms as our estimate for ϕ, the relative infectiousness of vaccinated persons, which assumes a linear relation between total *V. cholerae* output and transmission risk (Supplemental Table S7). However, the extent to which OCV limits infectivity is uncertain. Shedded bacterial density is unlikely to have one-to-one linear correspondence with hosts' infectivity because external factors influencing biofilm formation, vibriophage activity, the presence of aquatic copepods, and *V. cholerae* kinetics affect survival of *V. cholerae* in the environment [24]. Linear relations between bacterial shedding and infectiousness would additionally imply implausible variation in the reproductive number for asymptomatic and symptomatic cases [25]. Due to these sources of uncertainty, we conducted a sensitivity analysis where we assumed vaccine protection conferred between a 0.0097-fold reduction in transmission risk (i.e., greater than 99% protection against transmission, double the effect inferred from reductions described in [21]) to a 0.5-fold reduction in transmission risk (Table S7).

S2.5 Benefit to peacekeepers: reduction in disease risk

An additional benefit of chemoprophylaxis and OCV interventions is that these approaches reduce peacekeepers' risk for experiencing cholera symptoms. We quantified this individual-level benefit of participating in the interventions using previous estimates of the reductions in risk for cholera diarrhea associated with chemoprophylaxis and immunization.

S2.5.1 Antimicrobial chemoprophylaxis

Antimicrobial chemoprophylaxis confers two types of protection against cholera disease, first reducing an individual's probability for acquiring infection, and second reducing an individual's probability for experiencing symptoms if infection occurs. However, previous chemoprophylaxis studies have not measured the combined reduction in disease risk associated with these mechanisms of protection [32]. We therefore modeled the probability for disease in a peacekeeper receiving prophylaxis as the failure of both protective mechanisms. We use the previous estimate υ_{Abx} (Section S2.3) from a meta-analysis [32] for the probability that a cholera-exposed peacekeeper would develop infection given receipt of antimicrobials. We inferred protection against symptoms onset given infection using outcomes of a study [26] that compared the probability for diarrhea among asymptomatic-at-baseline *V. cholerae* carriers randomized to receive ciprofloxacin or placebo. We defined χ_{Abx} as the relative risk for symptoms given infection in a prophylaxed infected individual compared to an infected individual receiving placebo. Because 1 out of 15 ciprofloxacin recipients experienced disease in the trial in comparison to 6 out of 16 placebo recipients, we let

$$\chi_{\text{Abx, Infect}} = \frac{\mathbb{P}_{\text{Abx}}[\text{Symptoms}|\text{Infection}]}{\mathbb{P}_{\text{Base}}[\text{Symptoms}|\text{Infection}]} \sim \frac{\text{Beta}(1,14)}{\text{Beta}(6,10)} = 13.2\% \; [0.4\%,\, 74.6\%].$$

The total reduction in risk for disease in a peacekeeper receiving prophylaxis is thus $1 - \chi_{\text{Abx, Infect}}\nu_{\text{Abx}} = 95.5\% \; [70.4\%,\, 99.9\%]$.

S2.5.2 Oral cholera vaccination

In contrast to studies of antimicrobial chemoprophylaxis, a previous study directly estimated total reductions in disease risk among OCV recipients [21]. In the trial, 3 out of 9 vaccine recipients experienced cholera diarrhea, compared to 13 out of 15 placebo recipients. We thus defined the total reduction in disease risk as

$$1 - \chi_{\text{Vax}} = 1 - \frac{\mathbb{P}_{\text{Vax}}[\text{Symptoms}]}{\mathbb{P}_{\text{Base}}[\text{Symptoms}]} \sim 1 - \frac{\text{Beta}(3,6)}{\text{Beta}(13,2)} = 62.8\% \; [22.2\%,\, 90.0\%]$$

This protection against disease in the challenge trial was similar to estimates in larger field trials which measured direct effects [22,27].

S2.5.3 Combined chemoprophylaxis and immunization

As discussed previously, OCV does not appear to offer significant direct protection against cholera infection (Section S2.4). We therefore modeled disease in peacekeepers receiving both antimicrobial chemoprophylaxis and OCV as a clinical failure on three fronts: failure for antimicrobials to protect against (1) infection and (2) disease onset given infection, and (3) failure for OCV to protect against disease onset given infection. In the vaccine trial [21], 7 symptomatic cases occurred among 17 vaccine recipients who experienced infection,

in contrast to 13 symptomatic cases out of 15 placebo recipients (outcomes were pooled for the vaccines with and without B subunit due to perfect prediction in the arm receiving vibrio-only vaccine). Reduction in risk for disease onset given infection in the vaccine trial was estimated as

$$\begin{aligned} 1 - \chi_{\text{Vax, Infect}} &= 1 - \frac{\mathbb{P}_{\text{Vax}}[\text{Symptoms}|\text{Infection}]}{\mathbb{P}_{\text{Base}}[\text{Symptoms}|\text{Infection}]} \\ &\sim 1 - \frac{\text{Beta}(7, 10)}{\text{Beta}(13, 2)} = 52.8\% \ [21.4\%, \ 77.9\%], \end{aligned}$$

so that the total reduction in disease risk under a combined intervention was

$$1 - \nu_{\text{Abx}}\chi_{\text{Abx, Infect}}\chi_{\text{Vax, Infect}} = 98.0\% \ [84.9\%, \ 99.9\%].$$

S3 Model description

S3.1 Disease transitions

We formulated a compartmental model described by a system of ordinary differential equations tracking the infection status of populations comprising susceptible (S), exposed (E), infected (I), and recovered (R) individuals, as well as an environmental *V. cholerae* reservoir (B). While similar to other mathematical models for epidemic cholera, our model included several novel elements accounting for sources of uncertainty in previous models [25].

For the general population of Haiti, we assumed there was no previous exposure or immunity to cholera. We defined two transmission pathways by which susceptible individuals became infected. The first resulted from local transmission as defined in the Main Text, inducing new infections at the rate λ_L. The second resulted from exposure to environmental *V. cholerae* in the Artibonite watershed and induced new infections at the rate λ_W.

Upon infection, individuals progressed from the S to the E classes corresponding to asymptomatic (E_A) and symptomatic (E_S) clinical outcomes depending on the dose of *V. cholerae* to which they had been exposed [25,36]. During the E phase, individuals incubated infection latently prior to onset of asymptomatic or symptomatic shedding. Individuals progressed at the rate δ to the asymptomatic (I_A) and symptomatic (I_S) infectious classes, where $1/\delta$ = 1.55 d was the mean duration of the incubation period [11]. Asymptomatic infections lasted on average $1/\gamma_A$ = 5.09 d, resolving to an immune condition (R) in which individuals did not become re-infected [37,38]. Individuals who experienced disease died with probability $\zeta = 2.5\%$ according with case-fatality during the early phases of the Haiti epidemic in the Artibonite region [39]. Non-fatal infections resolved at the rate γ_D as individuals entered an asymptomatic convalescent state (I_C). The mean duration of symptoms among survivors was $1/\gamma_D$ = 3.32 d, while pathogen excretion continued on average $1/\gamma_C$ = 1.77

d following symptoms resolution [33,37,38,40–43]. Symptomatic cholera cases likewise recovered into the immune state (R).

During the early phases of the cholera outbreak, most deaths reportedly occurred in hospitals or other healthcare settings where decedents were less likely to contribute to transmission, while deaths within the community reportedly occurred within 12 hours (median) of symptoms onset and within as few as 2 hours [39]. Owing to this right-tailed shape of the time-to-death distribution, we accounted for reduced opportunities for transmission from decedents by modeling exits occurring from the I_S compartment at a net rate of $\gamma_D(1-\zeta)^{-1}$.

Last, we accounted for hyperinfectivity in freshly-passaged *V. cholerae* (B_1) in accordance with empirical findings [44, 45] and previous models [46]. We assumed newly-shedded *V. cholerae* in the B_1 compartment contributed to new infections at a rate elevated by the factor η. We modeled hyperinfectivity waning at the rate μ_1 so that *V. cholerae* subsequently persisted in a longer-term state (B_2) associated with lower infectiousness. We list model parameters and their sources in Tables 2 and 3, and illustrate the model schematically in Figure S1.

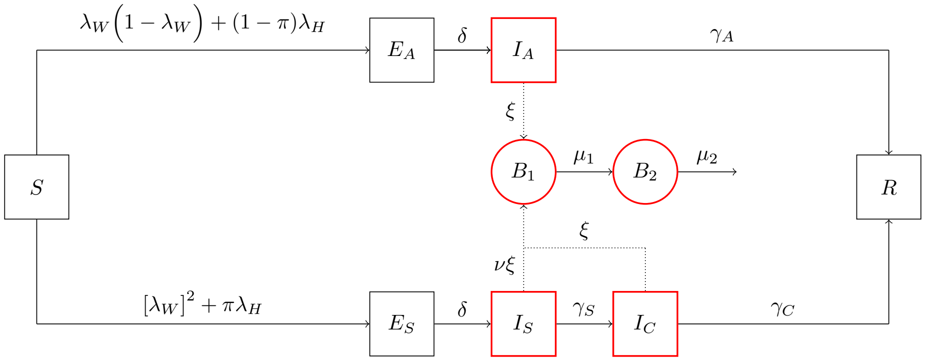

Figure S1: Infectious persons progressed to a latent phase preceding either asymptomatic or symptomatic *V. cholerae* shedding (square boxes). We modeled free-living *V. cholerae* shedded by persons who were directly exposed to the Artibonite River, which entered the environment in a short-lived hyperinfective state (circles).

S3.2 Force of infection

To account for transmission via contact with the Artibonite watershed, we let

$$\lambda_W = \frac{\beta_W(\eta B_1 + B_2)}{\beta_W(\eta B_1 + B_2) + \kappa}$$

where κ was the dose associated with a 50% probability for infection, and β_W was the rate individuals came into contact with waterborne *V. cholerae* (esti-

mated; Table 3). The approach allowed us to interpret B_1, B_2, and κ in terms of dosage rather than concentration. Previous models following [53] have defined B_1, B_2, and κ instead according to the concentration of *V. cholerae* in the environment. However, this more common approach results in uncertainty due to the fact that the volume of the infected water source is often unknown, and is not directly related to the modeled state variables [25,54].

Estimates for the cholera infectious dose have varied widely in volunteer challenge trials due to host-level factors as well as whether individuals ingested *V. cholerae* with or without bicarbonate buffer solution [25,36]. This uncertainty precluded interpretation of numerical values for κ in a model involving natural *V. cholerae* exposure in the environment. As a flexible alternative to previous models that sought to quantify *V. cholerae* exposure directly, we fixed κ as a numerical constant (0.1) and estimated β_W values corresponding to exposure levels requisite for inducing infections as they were observed in Haiti.

We modeled frequency-dependent local transmission in the population at large using the force of infection

$$\lambda_L = k\ln\left(1 + \frac{\beta_L(I_A + I_C + rI_S)}{k}\right)$$

so that the expected number of new cases individuals cause followed a negative binomial distribution over successive generations of infections. The constant k served as an index of disaggregation among infections [55]. Smaller values of k would reflect clustered transmission mediated by close interpersonal contact, which is a distinguishing feature of cholera epidemiology, while larger values ($k \to \infty$) would result in homogenous "mass-action" transmission within the population. We estimated k during the model calibration, as described in Section S4.

S3.3 Heterogeneous infectivity

Infectious individuals contributed to both the local and Artibonite-mediated transmission pathways by shedding *V. cholerae* into the surrounding environment. Stool volume for symptomatic individuals was modeled to be on average $\nu = 8.58$-fold greater than for asymptomatic individuals,

$$\nu = \left(\frac{3500 \text{ mL } (\textit{symptomatic})}{1/\gamma_D = 3.32 \text{ d}}\right)\left(\frac{1}{123 \text{ mL } (\textit{asymptomatic})}\right) = 8.58,$$

considering that symptomatic cases excreted on average 3500 mL of stool during their illness [36,42,47] compared to 123 mL per day among asymptomatic individuals [56]. This difference in infectiousness was consistent with previous models [52,57]. We assumed symptomatic individuals' contribution to local transmission was elevated by a factor $r = 1 + \text{Log}_{10}\nu$. Due to uncertainty in the relative infectiousness of asymptomatic and symptomatic persons in the context of

local transmission, we conducted sensitivity analyses assuming $r = 1 + \frac{1}{2}\text{Log}_{10}\nu$ and $r = 1 + 2\text{Log}_{10}\nu$.

Persons who came into direct contact with the Artibonite watershed for drinking, cleaning, and hygiene needs had the ability to induce new infections over long geographic distances by downstream hydrological transport. Consequently, we tracked their contributions to environmental *V. cholerae* in the Artibonite. Among persons who were directly exposed to this water source, we defined $\xi = 1$ d^{-1} (fixed; Table 1) as the rate of shedding *V. cholerae* into the environment during asymptomatic infection, so that symptomatic individuals contributed at the rate $\nu\xi$ d^{-1}.

S3.4 Clinical outcomes of infection

We accounted for dose dependence in cholera infection and symptoms by defining λ_W as both the rate at which individuals acquired infection and the conditional probability for experiencing symptoms given that infection occurred. Therefore, new symptomatic infections arose at the rate λ_W^2, while new asymptomatic infections arose at the rate $(1 - \lambda_W)\lambda_W$.

Due to heterogeneity in the exposures mediating local transmission, we defined the probability for symptoms among persons acquiring infection via this pathway as a time-invariant parameter π, which we estimated with the aid of a prior distribution inferred from a challenge study in immunologically-naive volunteers [51] and a post-outbreak serological study [49] in the Artibonite region of Haiti (Table 2). We detail the derivation of prior distributions in Section S4.2.

S3.5 Peacekeeping compartments

We defined I_P as the number of infectious peacekeepers in the absence of biomedical interventions, and assumed peacekeepers contributed to new cases via contamination of the Artibonite watershed. In the context of a vaccine intervention, all peacekeepers received OCV and, if infectious, belonged to the compartment $I_P^{(V)}$, in which they shed at the reduced rate $\phi\xi$ owing to vaccine-induced immunity (Section S2.4). In the context of antimicrobial prophylaxis, infectious peacekeepers belonged to $I_P^{(\text{Abx})}$ and recovered at the accelerated rate γ_{Abx} (Section S2.3). We varied ϕ and γ_{Abx} in sensitivity analyses.

S3.6 Population structure and migration

We distributed the population of Haiti across several geographically-defined patches. The first (A) comprised the eight severely-affected Artibonite-adjacent communes situated downstream from the MINUSTAH base (Mirebalais, St-Marc, Dessalines, Desdunes, Grande Saline, L'Estère, Petite-Rivière-de-l'Artibonite,

Verrettes) that experienced cases in the first stage of the outbreak, with an aggregate population of 879,644 [13,48].

Several upstream Artibonite-adjacent communes located between Mirebalais and the Artibonite Delta (Saut d'Eau: 0 cases; Boucan Carre: 0 cases; La Chapelle: 2 cases; Gonaïves: 1 imported case) did not have settlements along the Artibonite banks, or were served by water from a different river network flowing from the North (Gonaïves only) [13] and consequently did not experience a severe early epidemic as the consequence of transmission via the Artibonite River. We therefore included these latter four communes along with all other communes in Haiti within a separate patch (A^C) having an aggregate population of 9,043,599 [48].

Within the river-adjacent communes that experienced the initial outbreak, we defined a subpatch (W) comprising individuals who came into contact with the Artibonite watershed and acquired or transmitted infection via this exposure. We distinguished these persons from the remaining population (subpatch W^C) who neither acquired not transmitted infection via Artibonite exposure. Rice cultivation in fields irrigated by river-fed channels was a dominant economic activity in the Lower Artibonite, and men working in the rice fields were the predominant at-risk group for cholera admissions in this region during the early phase of the outbreak [13,49,58]. To account for temporal intermittency in exposure to the water source, due for instance to itinerant or migratory labor from villages situated further from the Artibonite River, we modeled movement in and out of the Artibonite-exposed subpatch at the following rates:

$$\frac{\mathrm{d}N^{(W)}}{\mathrm{d}t} = \omega \left[\frac{N_0^{(W)}}{N_0^{(A)}} N^{(W^C)} - \frac{N_0^{(W^C)}}{N_0^{(A)}} N^{(W)} \right]$$
$$\frac{\mathrm{d}N^{(W^C)}}{\mathrm{d}t} = \omega \left[\frac{N_0^{(W^C)}}{N_0^{(A)}} N^{(W)} - \frac{N_0^{(W)}}{N_0^{(A)}} N^{(W^C)} \right]$$

Here the relative sizes of the (W) and (W^C) subpatches determined the probability for individuals to enter either, while ω (estimated; Table 3) gave the total rate of movement into or out of the subpatches. We assumed that mobility was restricted for persons experiencing cholera symptoms, so that these individuals did not migrate between subpatches while in the I_S state. For shorthand, the functions

$$m_W(x) = \omega \left[\frac{N_0^{(W)}}{N_0^{(A)}} x^{(W^C)} - \frac{N_0^{(W^C)}}{N_0^{(A)}} x^{(W)} \right]$$
$$m_{W^C}(x) = \omega \left[\frac{N_0^{(W^C)}}{N_0^{(A)}} x^{(W)} - \frac{N_0^{(W)}}{N_0^{(A)}} x^{(W^C)} \right]$$

describe migration between the (W) and (W^C) subpatches for any disease state $x|x \neq I_S$.

S3.7 Model equations

The modeled transitions in the populations of each compartment were described using a system of ordinary differential equations. For the populations within the (A) patch:

$$\frac{\mathrm{d}S^{(W)}}{\mathrm{d}t} = -(\lambda_L + \lambda_W)S^{(W)} + m_W(S)$$

$$\frac{\mathrm{d}S^{(W^C)}}{\mathrm{d}t} = -\lambda_L S^{(W^C)} + m_{W^C}(S)$$

$$\frac{\mathrm{d}E_A^{(W)}}{\mathrm{d}t} = \left[(1-\pi)\lambda_L + (1-\lambda_W)\lambda_W\right] S^{(W)} - \delta E_A^{(W)} + m_W(E_A)$$

$$\frac{\mathrm{d}E_A^{(W^C)}}{\mathrm{d}t} = (1-\pi)\lambda_L S^{(W^C)} - \delta E_A^{(W^C)} + m_{W^C}(E_A)$$

$$\frac{\mathrm{d}E_S^{(W)}}{\mathrm{d}t} = (\pi\lambda_L + \lambda_W^2)S^{(W)} - \delta E_S^{(W)} + m_W(E_S)$$

$$\frac{\mathrm{d}E_S^{(W^C)}}{\mathrm{d}t} = \pi\lambda_L S^{(W^C)} - \delta E_S^{(W^C)} + m_{W^C}(E_S)$$

$$\frac{\mathrm{d}I_A^{(W)}}{\mathrm{d}t} = \delta E_A^{(W)} - \gamma_A I_A^{(W)} + m_W(I_A)$$

$$\frac{\mathrm{d}I_A^{(W^C)}}{\mathrm{d}t} = \delta E_A^{(W^C)} - \gamma_A I_A^{(W^C)} + m_{W^C}(I_A)$$

$$\frac{\mathrm{d}I_S^{(W)}}{\mathrm{d}t} = \delta E_S^{(W)} - \frac{\gamma_D}{1-\zeta} I_S^{(W)}$$

$$\frac{\mathrm{d}I_S^{(W^C)}}{\mathrm{d}t} = \delta E_S^{(W^C)} - \frac{\gamma_D}{1-\zeta} I_S^{(W^C)}$$

$$\frac{\mathrm{d}I_C^{(W)}}{\mathrm{d}t} = \gamma_D I_S^{(W)} - \gamma_C I_C^{(W)} + m_W(I_C)$$

$$\frac{\mathrm{d}I_C^{(W^C)}}{\mathrm{d}t} = \gamma_D I_S^{(W^C)} - \gamma_C I_C^{(W^C)} + m_{W^C}(I_C)$$

$$\frac{\mathrm{d}R^{(W)}}{\mathrm{d}t} = \gamma_A I_A^{(W)} + \gamma_C I_C^{(W)} + m_W(I_C)$$

$$\frac{\mathrm{d}R^{(W^C)}}{\mathrm{d}t} = \gamma_A I_A^{(W^C)} + \gamma_C I_C^{(W^C)} + m_{W^C}(I_C)$$

From any starting population of infectious peacekeepers,

$$\frac{\mathrm{d}I_P}{\mathrm{d}t} = -\gamma_A I_P$$

$$\frac{\mathrm{d}I_P^{(V)}}{\mathrm{d}t} = -\gamma_A I_P^{(V)}$$

$$\frac{\mathrm{d}I_P^{(\mathrm{Abx})}}{\mathrm{d}t} = -\gamma_{\mathrm{Abx}} I_P^{(\mathrm{Abx})}$$

$$\frac{\mathrm{d}I_P^{(\mathrm{Abx},V)}}{\mathrm{d}t} = -\gamma_{\mathrm{Abx}} I_P^{(\mathrm{Abx},V)}$$

Thus, we assumed no ongoing transmission at the MINUSTAH base. For environmental *V. cholerae* in the Artibonite watershed,

$$\frac{\mathrm{d}B_1}{\mathrm{d}t} = \xi \left(I_P + \phi(I_P^{(V)} + I_P^{(V,\mathrm{Abx})}) + I_A^{(W)} + \nu I_S^{(W)} + I_C^{(W)} \right) - \mu_1 B_1$$

$$\frac{\mathrm{d}B_2}{\mathrm{d}t} = \mu_1 B_1 - \mu_2 B_2$$

For the remaining population outside the Artibonite-adjacent communes (A^C):

$$\frac{\mathrm{d}S^{(A^C)}}{\mathrm{d}t} = -\lambda_L S^{(A^C)}$$

$$\frac{\mathrm{d}E_A^{(A^C)}}{\mathrm{d}t} = (1-\pi)\lambda_L S^{(A^C)} - \delta E_A^{(A^C)}$$

$$\frac{\mathrm{d}E_S^{(A^C)}}{\mathrm{d}t} = \pi \lambda_L S^{(A^C)} - \delta E_S^{(A^C)}$$

$$\frac{\mathrm{d}I_A^{(A^C)}}{\mathrm{d}t} = \delta E_A^{(A^C)} - \gamma_A I_A^{(A^C)}$$

$$\frac{\mathrm{d}I_S^{(A^C)}}{\mathrm{d}t} = \delta E_S^{(A^C)} - \frac{\gamma_D}{1-\zeta} I_S^{(A^C)}$$

$$\frac{\mathrm{d}I_C^{(A^C)}}{\mathrm{d}t} = \gamma_D I_S^{(A^C)} - \gamma_C I_C^{(A^C)}$$

$$\frac{\mathrm{d}R^{(A^C)}}{\mathrm{d}t} = \gamma_A I_A^{(A^C)} + \gamma_C I_C^{(A^C)}$$

S3.8 Stochastic implementation

We used the Gillespie algorithm [59] to implement the model stochastically. We defined the time elapsing between any two events as an exponentially-distributed random variable, with a rate parameter given by the sum of the rates for all transitions in the model at that time point. To determine which event occurred after a time step, we sampled probabilistically from the vector of rates and updated the time accordingly. We populated intervention efficacies in model realizations using Monte Carlo draws from the reported or inferred distributions of effect size estimates.

The computationally-intensive nature of this approach precluded conducting multiple simulations for each sampled vector of parameters. Consequently we generated 95% credible intervals in the probability of a case via bootstrap resampling. We used 5000 realizations of the model at randomly-sampled parameter sets, determining this approach provided 80% power for detecting a reduction by 5% in the probability of a case at the 95% significance level.

S3.9 Reproductive numbers

We derived basic reproductive numbers ($\mathcal{R}_0$) using the next-generation approach as described by van den Driessche and Watmough [60]. Considering a *V. cholerae*-free equilibrium, the rates of change in the compartments of each patch were

$$\dot{\mathbf{X}} = \left.\begin{bmatrix} \dot{E}_A \\ \dot{E}_S \\ \dot{I}_A \\ \dot{I}_S \\ \dot{I}_C \\ \dot{B}_1 \\ \dot{B}_2 \end{bmatrix}\right|_{S=N,x_i=0} = \mathscr{F}_W(X) + \mathscr{F}_L(X) - \mathscr{V}(X)$$

New infections occurred at the rates

$$\mathscr{F}_W = \begin{bmatrix} (1-\lambda_W)\lambda_W S^{(W)} \\ \lambda_W^2 S^{(W)} \\ 0 \\ \vdots \\ 0 \end{bmatrix}$$

for the pathway mediated by Artibonite exposure, and

$$\mathscr{F}_L(X) = \begin{bmatrix} (1-\pi)\lambda_L S \\ \pi\lambda_L S \\ 0 \\ \vdots \\ 0 \end{bmatrix}$$

for the local transmission pathway. All other transitions occurred at the rates

$$\mathscr{V} = \begin{bmatrix} \delta E_A \\ \delta E_S \\ \gamma_A I_A - \delta E_A \\ \gamma_D (1-\zeta)^{-1} I_S - \delta E_S \\ \gamma_C I_C - \gamma_D I_S \\ \mu_1 B_1 - \xi(I_A + \nu I_S + I_C) \\ \mu_2 B_2 - \mu_1 B_1 \end{bmatrix}$$

Evaluating the Jacobians at the *V. cholerae*-free equilibrium,

$$\mathbf{F}_W = \frac{\mathrm{d}\mathscr{F}_W(i)}{\mathrm{d}X(j)} = \begin{bmatrix} 0 & \cdots & 0 & \eta\beta_W(1-\frac{\eta\beta_W}{\kappa})\kappa^{-1}S^{(W)} & \beta_W(1-\frac{\beta_W}{\kappa})\kappa^{-1}S^{(W)} \\ \vdots & \ddots & \vdots & \eta^2\beta_W^2\kappa^{-2}S^{(W)} & \beta_W^2\kappa^{-2}S^{(W)} \\ & & & 0 & 0 \\ & & & \vdots & \vdots \\ 0 & \cdots & & 0 & 0 \end{bmatrix},$$

$$\mathbf{F}_L = \frac{\mathrm{d}\mathscr{F}_P(i)}{\mathrm{d}X(j)} = \begin{bmatrix} 0 & 0 & (1-\pi)\beta_L & (1-\pi)r\beta_L & (1-\pi)\beta_L & 0 & 0 \\ & & \pi\beta_L & \pi r\beta_L & \pi\beta_L & & \\ \vdots & & 0 & 0 & 0 & & \vdots \\ & & \vdots & \vdots & \vdots & & \\ 0 & 0 & 0 & 0 & 0 & 0 & 0 \end{bmatrix},$$

and

$$\mathbf{V} = \frac{\mathrm{d}\mathscr{V}(i)}{\mathrm{d}X(j)} = \begin{bmatrix} \delta & 0 & & & & \cdots & 0 \\ 0 & \delta & 0 & & & & \vdots \\ -\delta & 0 & \gamma_A & 0 & & & \\ 0 & -\delta & 0 & \frac{\gamma_D}{(1-\zeta)} & 0 & & \\ 0 & 0 & 0 & -\gamma_D & \gamma_C & 0 & \\ 0 & 0 & -\xi & -\nu_S\xi & -\xi & \mu_1 & 0 \\ 0 & 0 & 0 & 0 & 0 & -\mu_1 & \mu_2 \end{bmatrix}.$$

Defining $\mathcal{R}_0^W$ as the dominant eigenvalue $\rho\left(\mathbf{F}_W\mathbf{V}^{-1}\right)$ and correspondingly $\mathcal{R}_0^L = \rho\left(\mathbf{F}_P\mathbf{V}^{-1}\right)$, we obtained

$$\mathcal{R}_0^W = \beta_W \frac{\Big(\gamma_C\gamma_D(1-\zeta)\kappa(\mu_1+\eta\mu_2) + \beta_W(\mu_1+\eta^2\mu_2)\big(\gamma_A(\gamma_D\nu_S\gamma_C) - \gamma_C\gamma_D(1-\zeta)\big)\Big)\xi}{\gamma_A\gamma_C\gamma_D(1-\zeta)\kappa^2\mu_1\mu_2} N_0^{(W)}$$

and

$$\mathcal{R}_0^L = \beta_L\left(\frac{1-\pi}{\gamma_A} + \pi\frac{\gamma_C r + \gamma_D}{\gamma_C\gamma_D(1-\zeta)}\right).$$

For any individual in Haiti, the reproductive number was the sum of all transmission expected to occur via the environmental and local pathways. Aggregating reproductive numbers geographically, we considered an $\mathcal{R}_0^A$ for the Artibonite-adjacent communes and a nation-wide $\mathcal{R}_0$ accounting for all patches (j). Because the rate of movement into the (W) subpatch was low $(\omega N_0^{(W)}/N_0^{(A)} < 10^{-3})$ and symptomatic cases, which contributed overwhelmingly to $\mathcal{R}_0^W$, were

assumed not to be mobile, migration had negligible impact (Table 3). Consequently,

$$\mathcal{R}_0^A \approx \mathcal{R}_0^L + \frac{N^{(W)}}{N_0^{(A)}}\mathcal{R}_0^W$$

$$\mathcal{R}_0 \approx \mathcal{R}_0^L + \frac{N_0^{(W)}}{\sum_J N_0^{(j)}}\mathcal{R}_0^W.$$

S4 Model calibration

S4.1 Parameter estimation and multimodel inference

We identified fixed values for observable clinical parameters via a literature survey, and fitted remaining epidemiological parameters by calibrating model output to daily suspected and confirmed cholera cases in ambulatory patients from 16 October to 5 November 2010 in Haiti. We list fixed parameters in Table 1 along with their numerical values and literature sources, and estimated parameters in Table 2 with prior distributions (Section S4.2) used in the fitting procedure.

We calibrated the model by exploring parameter distributions under scenarios where the epidemic was initiated by either one, two, or three infected peacekeepers, noting that the status-quo probability for more than three peacekeepers to have arrived infected was negligible ($< 10^{-4}$) even assuming the highest background incidence rate in Nepal (Table S3). We predicted qualitatively different epidemic dynamics under these scenarios with respect to the relative importance of hydrological and locally-mediated *V. cholerae* transmission (Table 5). Consequently, we explored the parameter state space conditioned on the number of arriving infected peacekeepers being equal to one, two, or three in separate procedures. We then pooled model-based estimates across their marginal distributions for $X_0 = \{1, 2, 3\}$, weighting according to the probabilities $\mathbb{P}(X_0 = 1, 2, 3)$ (Table S3).

S4.2 Prior distributions

We derived an informative Beta-distributed prior for π, the probability for individuals to experience symptoms following infection via local transmission, using findings from a serological survey [49] conducted in the Artibonite region of Haiti in March-April 2011 and a challenge study among immunologically-naive volunteers [51]. The serosurvey contributed the majority of observations ($n = 948$, among whom 242 experienced cholera; for the challenge study $n = 127$, among whom 21 experienced cholera). We defined $\alpha = 263$ and $\beta = 812$ as the number of infected individuals who experienced symptomatic and asymptomatic infections, respectively, so that the prior was centered at 24.4% [21.9%, 27.1%].

Because the case series used for evaluating the model's likelihood presented data from symptomatic cases only, the informative prior was critical as a basis for statistical identification of the symptom probability. Sensitivity analyses considering weaker priors for the symptom probability revealed that estimates for this parameter were highly correlated with estimates of the local transmission rate. Using an informative prior resolved the identifiability problem between π and β_L without drastically altering the mean of the posterior distribution (Supplemental Figure S2).

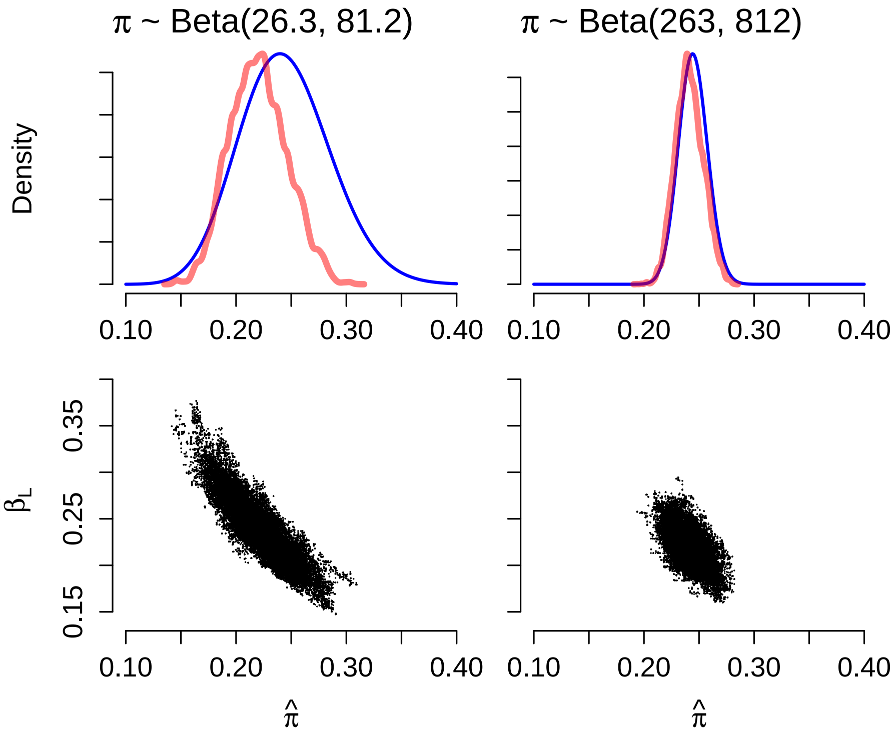

Figure S2: The posterior distribution (pink) of the symptom probability (π) closely matched the prior distribution (blue; top panel). Use of an informative prior resolved identifiability problems affecting the symptom probability and the local transmission rate β_L.

We defined a uniform prior distribution bounded by 0.1 and 0.6 for β_L, corresponding to an $\mathcal{R}_0^L$ estimate between roughly 0.6 and 3.6 consistent with previous estimates for epidemic cholera in Haiti and elsewhere [25,52]. As per previous models limiting the population with direct river contact [52], we assumed the population exposed to *V. cholerae* in the Artibonite watershed would be between 1/1000 and 1/10 of the total population for these communes (879,644)

based on maps showing the population density to be 76–200 persons per square kilometer living within one km of the Artibonite banks [13] along a roughly 100-km stretch of Artibonite-fed waterways. We used non-informative priors for all other parameters (Table 3).

S4.3 Markov chain Monte Carlo sampling

We calibrated the model in its continuous-time deterministic formulation by comparing model-predicted ($\hat{Y}$) and observed (Y) cases in the Artibonite-adjacent and non-adjacent communes using a Bayesian framework. We computed model-predicted incident symptomatic cases on day t in each population (j) as

$$\hat{Y}_t = \int_{t-1}^{t} \delta E_S^{(j)}(t)\mathrm{d}t$$

We defined the vectors of case reports from the Artibonite-adjacent and non-adjacent communes as $\mathbf{y}^{(A)}$ and $\mathbf{y}^{(A^C)}$, respectively, which we digitized from Figure 2 of [13].

We used a Metropolis-Hastings approach to sample from the posterior distribution of the parameter vector

$$\theta = (\beta_W, \beta_L, k, \pi, \omega, N^{(W)}/N^{(A)})$$

based on the joint likelihood of the data vectors $\mathbf{y}^{(1)}$ and $\mathbf{y}^{(2)}$ given model-predicted output $\hat{\mathbf{y}}^{(A)}, \hat{\mathbf{y}}^{(A^C)}|\theta$. We computed the likelihood of daily incident case reports in each population by modeling

$$Y_t^{(j)} \sim \text{Pois}\left(\hat{Y}_t^{(j)}|\theta\right),$$

so that, for each day, $\mathcal{L}(\theta|Y_t^{(A)}, Y_t^{(A^C)})$ was the product of the Poisson densities

$$f_{\hat{Y}_t^{(A)}|\theta}\left(Y_t^{(A)}\right) f_{\hat{Y}_t^{(A^C)}|\theta}\left(Y_t^{(A^C)}\right).$$

Accounting for a joint prior density $P(\theta)$, the posterior probability for any θ was

$$P(\theta|\hat{\mathbf{y}}^{(A)}, \hat{\mathbf{y}}^{(A^C)}) = P(\theta) \prod_{t=1}^{\max(t)} \mathcal{L}\left(\theta|Y_t^{(A)}, Y_t^{(A^C)}\right).$$

We used the prior probabilities presented in Table 3 and sampled using three independent parallel chains initiated at randomly-chosen values with 50,000 iterations, discarding the first 10,000 as burn-in. We updated the parameters in blocks, choosing between one and six of the parameters fully at random for updating at each step. We identified proposal distributions that yielded between 30% and 70% overall acceptance rates following burn-in, and did not vary proposal distributions during state space exploration. We assessed chain

convergence to the stationary distribution visually and using the potential scale reduction factor (PSRF) [61]. In comparison to conventional cut-offs specifying the PSRF should be below 1.1 or 1.2 [62], this value was 1.04 or lower across all instances assuming differing initial conditions and derivations of r. We report fitted parameters under the differing assumptions in Table S15.

S4.4 Timing of the first case

Because surveillance for suspected cholera cases began October 16 [13], the timing of the initial case was not captured in the dataset against which we calibrated the model. This timing was subject to considerable stochastic variation across realizations of the model [95% CI October 11 to 20]. We illustrate the distribution of the timing of the first case across realizations of the model in Figure S3.

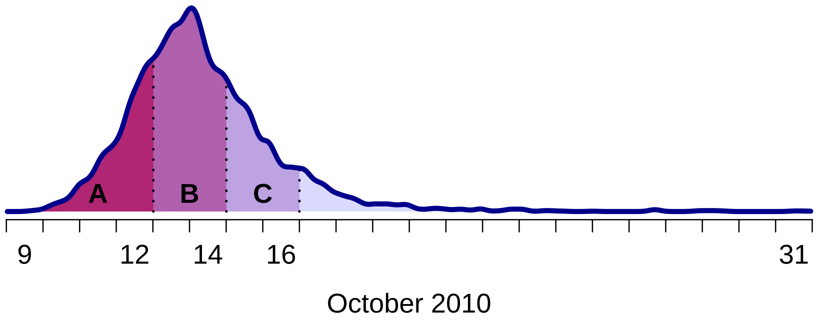

Figure S3: Timing of the first case. We illustrate the probability distribution describing the first date the model predicts a symptomatic case (density on Y axis). The dates marked on the timeline include October 9 (arrival of peacekeepers at the MINUSTAH base), October 12 (first case documented by [63]), October 14 (first culture-confirmed cases identified in an outbreak investigation [13]), and October 16 (surveillance initiated [13]). The model predicts a case on or before October 12, 14, and 16 in 24%, 71%, and 92% of simulations, respectively, here represented by the areas in the regions A, A+B, and A+B+C. The illustrated probability distribution is weighted according to an assumed background incidence rate of 1.8 cases per 1000 person-years at risk (PYAR) in Nepal, and does not differ significantly assuming between 0.5 and 10.0 cases per 1000 PYAR.

.

S5 References

1. Cravioto A, Lanata CF, Latagne DS, Balakrish Nair G. Final report of the independent panel of experts on the cholera outbreak in Haiti. 2011. Available: http://bit.ly/1yGscKw.

2. Ali M, Lopez AL, You Y, et al. The global burden of cholera. Bull World Health Organ. 2012;90: 209–18.

3. Spira WM, Khan MU, Saeed YA, Sattar MA. Microbiological surveillance of intra-neighbourhood El Tor cholera transmission in rural Bangladesh. Bull World Health Organ. 1980;58: 731–40.

4. Bart KJ, Huq Z, Khan M, Mosley WH, Nuruzzaman MD, Kibriya AKMG. Seroepidemiologic studies during a simultaneous epidemic of infection with El Tor Ogawa and Classical Inaba *Vibrio cholerae.* J Infect Dis. 1970;121: S17-S24.

5. Woodward WE, Mosley WH. The spectrum of cholera in rural Bangladesh II: Comparison of El Tor Ogawa and Classical Inaba infection. Am J Epidemiol. 1972;96: 342–51.

6. Tamayo JF, Mosley WH, Alvero MG, et al. Studies of cholera El Tor in the Philippines: 3. Transmission of infection among household contacts of cholera patients. Bull World Health Organ. 1965;33: 645–9.

7. Glass RI, Holmgren J, Haley CE, et al. Predisposition for cholera of individuals with O blood group: possible evolutionary significance. Am J Epidemiol. 1985;121: 791–6.

8. Harris JB, Khan AI, LaRocque RC, et al. Blood group, immunity, and risk of infection with *Vibrio cholerae* in an area of endemicity. Infect Immun. 2005;73: 7422–7.

9. Viechtbauer W. Conducting meta-analyses in R with the metafor package. J Stat Softw. 2010;36: 1–48.

10. General and Social Characteristics Tables. National Population and Housing Census 2011. Government of Nepal Central Bureau of Statistics. 2014. Available: http://bit.ly/1CJbzON.

11. Azman AS, Rudolph KE, Cummings DA, Lessler J. The incubation period of cholera: a systematic review. J Infect. 2013;66: 432–8.

12. Weil AA, Begum Y, Chowdhury F, et al. Bacterial shedding in household contacts of cholera patients in Dhaka, Bangladesh. Am J Trop Med Hyg. 2014;91: 738–42.

13. Piarroux R, Barrais R, Faucher B, et al. Understanding the cholera epidemic, Haiti. Emerg Infect Dis. 2011;17: 1161–8.

14. Alam M, Hasan NA, Sultana M, et al. Diagnostic limitations to accurate diagnosis of cholera. J Clin Microbiol. 2010;8: 3918–22.

15. Dick MH, Guillerm M, Moussy F, Chaignat CL. Review of two decades of cholera diagnostics—How far have we really come?. PLoS Negl Trop Dis. 2012;6: e1845.

16. Fact sheet: United Nations follow-up to the recommendations of the Independent Panel of Experts on the cholera outbreak in Haiti. United Nations. 2014. Available: http://bit.ly/1CMwnpC.

17. Bhuiyan NA, Qadri F, Faruque ASG, et al. Use of dipsticks for rapid diagnosis of cholera caused by *Vibrio cholerae* O1 and O139 from rectal swabs. J Clin Microbiol. 2003;41: 3939–41.

18. Chakraborty S, Alam M, Scobie HM, Sack DA. Adaptation of a simple dipstick test for detection of *Vibrio cholerae* O1 and O139 in environmental water. Front Microbiol. 2013;4: 320.

19. Nelson EJ, Nelson DS, Salam MA, Sack DA. Antibiotics for both moderate and severe cholera. N Engl J Med. 2011;364: 5–7.

20. McCormack WM, Chowdhury AM, Jahangir N, Fariduddin Ahmed AB, Mosley WH. Tetracycline prophylaxis in families of cholera patients. Bull World Health Organ. 1968;38: 787–92.

21. Black RE, Levine MM, Clements ML, Young CR, Svennerholm AM, Holmgren J. Protective efficacy in humans of killed whole-vibrio oral cholera vaccine with and without the B subunit of cholera toxin. Infect Immun. 1987;55: 1116–20.

22. Sanchez JL, Vasquez B, Begue RE, et al. Protective efficacy of oral whole-cell/recombinant-B-subunit cholera vaccine in Peruvian military recruits. Lancet. 1994;344: 1273–6.

23. Morris JG, Losonsky GE, Johnson JA, et al. Clinical and immunologic characteristics of *Vibrio cholerae* O139 Bengal infection in North American volunteers. J Infect Dis 1995;171: 903–8.

24. de Magny GC, Murtugudde R, Sapiano MRP, et al. Environmental signatures assocaited with cholera epidemics. Proc Natl Acad Sci U S A. 2008;105: 17676–81.

25. Grad YH, Miller JC, Lipsitch M. Cholera modeling: challenges to quantitative analysis and predicting the impact of interventions. Epidemiology (Cambridge). 2012;23: 523–30.

26. Echevarria J, Seas C, Carrillo C, Mostorino R, Ruiz P, et al. Efficacy and tolerability of ciprofloxacin prophylaxis in adult household contacts of patients with cholera. Clin Infect Dis 1995;20: 1480–4.

27. Ali M, Emch M, von Seidelin L, et al. Herd immunity conferred by killed oral cholera vaccines in Bangladesh: a reanalysis. Lancet 2005;366: 44–9.

28. Date KA, Vicari A, Hyde TB, et al. Considerations for oral cholera vaccine use during outbreak after earthquake in Haiti, 2010–2011. Emerg Infect Dis. 2011;17: 2105–12.

29. Harris JR, Cavallaro EC, de Nóbrega AA, et al. Field evaluation of Crystal VC rapid dipstick test for cholera during a cholera outbreak in Guinea-Bissau. Trop Med Int Health. 2009;14: 1117–21.

30. Mukherjee P, Ghosh S, Ramamurthy T, et al. Evaluation of a rapid immunochromatographic dipstick kit for diagnosis of cholera emphasizes its outbreak utility. Jpn J Infect Dis. 2010;63: 234–8.

31. Ley B, Khatib AM, Thriemer K, et al. Evaluation of a rapid dipstick (Crystal VC) for the diagnosis of cholera in Zanzibar and a comparison with previous studies. PLoS ONE. 2012;7: e36930.

32. Reveiz L, Chapman E, Ramon-Pardo P, et al. Chemoprophylaxis in contacts of patients with cholera: systematic review and meta-analysis. PLoS ONE. 2011;6: e27060.

33. Leibovici-Kalter Y, Paul M, Salam MA. Antimicrobial drugs for treating cholera. Cochrane Database Syst Rev. 2014. Available: http://bit.ly/1lYIWX8.

34. Sen Gupta PG, Sircar BK, Mondal S, et al. Effect of doxycycline on transmission of *Vibrio cholerae* infection among family contacts of cholera patients in Calcutta. Bull World Health Organ. 1978;56: 323–6.

35. Deb BC, Sen Gupta PG, De SP, Sil J, Sikdar SN, Pal SC. Effect of sulfadoxine on transmission of *Vibrio cholerae* infection among family contacts of cholera patients in Calcutta. Bull World Health Organ. 1976;54: 3504–9.

36. Cash RA, Music SI, Libonati JP, et al. Response of man to infection with *Vibrio cholerae*: I. Clinical, serologic, and bacteriologic responses to a known inoculum. J Infect Dis. 1974;129: 45–52.

37. Dutta D, Bhattacharya SK, Bhattacharya MK, et al. Efficacy of norfloxacin and doxcycycline for treatment of *Vibrio cholerae* O139 infection. J Antimicrob Chemother. 1996;37: 575–81.

38. Alam NH, Ashraf H, Khan WA, Karim MM, Fuchs GJ. Efficacy and tolerability of racecadotril in the treatment of cholera in adults: a double blind, randomized, controlled clinical trial. Gut. 2003;52: 1419–23.

39. Update: outbreak of cholera—Haiti, 2010. Morbid Mortal Wkly Rep. 2010;59: 1586–90.

40. Patra FC, Majumder RN, Eeckels R, Desjeux JF, Mahalanabis D. Sacolene in cholera: a double blind randomized controlled trial. Scand J Infect Dis. 1999;31: 151–4.

41. Rabbani GH, Islam MR, Butler T, Shahrier M, Alam K. Single-dose treatment of cholera with furszolidone or tetracycline in a double-blind randomized trial. Antimicrob Ag Chemother. 1989;33: 1447–50.

42. Lindenbaum J, Greenough WB, Islam MR. Antibiotic therapy of cholera. Bull World Health Organ. 1967;36: 871–83.

43. Hossain MS, Salam MA, Rabbani GH, Kabir I, Biswas R, Mahalanabis D. Tetracycline in the treatment of severe cholera due to *Vibrio cholerae* O139 Bengal. J Health Pop Nutr. 2011;20: 18–25.

44. Merrell DS, Butler SM, Qadri F, et al. Host-induced epidemic spread of the cholera bacterium. Nature. 2002;417: 642–5.

45. Alam A, LaRocque RC, Harris JB, et al. Hyperinfectivity of human-passaged *Vibrio cholerae* can be modeled by growth in the infant mouse. Infect Immun. 2005;73: 6674–9.

46. Hartley DM, Glenn Morris J, Smith DL. Hyperinfectivity: a critical element in the ability of *V. cholerae* to cause epidemics?. PLoS Med. 2005;3: e7.

47. Sack DA, Tacket CO, Cohen MB, et al. Validation of a volunteer model of cholera with frozen bacteria as the challenge. Infect Immun. 1998;66: 1968–72.

48. Haiti 2009 population data, section estimates, stratified by sex and age. Centers for Disease Control and Prevention 2012. Available: http://1.usa.gov/1AVcMnd.

49. Jackson BR, Talkington DF, Pruckler JM, et al. Seroepidemiologic survey of epidemic cholera in Haiti to assess spectrum of illness and risk factors for severe disease. Am J Trop Med Hyg. 2013;89: 654–64.

50. Tacket CO, Losonsky G, Nataro JP, et al. Initial clinical studies of CVD112 *Vibrio cholerae* O139 live oral vaccine: safety and efficacy against experimental challenge. J Infect Dis. 1995;172: 883–6.

51. Clements ML, Levine MM, Young CR, et al. Magnitude, kinetics, and duration of vibriocidal antibody responses in North Americans after ingestion of *Vibrio cholerae*. J Infect Dis. 1982;145: 465–73.

52. Chao DL, Halloran ME, Longini IM. Vaccination strategies for epidemic cholera in Haiti with implications for the developing world. Proc Natl Acad Sci U S A. 2011;180: 7081–5.

53. Codeço CT. Endemic and epidemic dynamics of cholera: the role of the aquatic reservoir. BMC Infect Dis. 2001;1.

54. Pascual M, Bouma MJ, Dobson AP. Cholera and climate: revisiting the quantitative evidence. Microb Infect. 2002;4: 237–45.

55. McCallum H, Barlow N, Hone J. How should pathogen transmission be modeled?. Trends Ecol Evol. 2001;16: 295–300.

56. Rendtorff RC, Kashgarian M. Stool patterns of healthy adult males. Dis Colon Rectum. 1967;10: 222–8.

57. Longini IM, Nizam A, Ali M, Yunus M, Shenvi N, Clemens JD. Controlling endemic cholera with oral vaccines. PLoS Med. 2007;4: e336.

58. Ernst S, Weinrobe C, Bien-Aime C, Rawson I. Cholera management and prevention at Hopital Albert Schweitzer, Haiti. Emerg Infect Dis. 2011;17: 2155–7.

59. Gillespie DT. A general method for numerically simulating the stochastic time evolution of coupled chemical reactions. J Comp Phys. 1976;22: 403–34.

60. van den Driessche P, Watmough J. Reproduction numbers and sub-threshold endemic equilibria for compartmental models of disease transmission. Math Biosci. 2002;180: 29–48.

61. Gelman A, Rubin DB. Inference from iterative simulation using multiple sequences. Stat Sci 1992;7: 475–72.

62. Gellman A, Carlin JB, Stern HS, Rbin DB. Bayesian data analysis. 1995; New York: Chapman & Hall.

63. Ivers LC, Walton DA. The “first” case of cholera in Haiti: lessons for global Health. Am J Trop Med Hyg 2012;86: 36–8.
